# Synthetic bacterial community derived from a desert rhizosphere confers salt stress resilience to tomato in the presence of a soil microbiome

**DOI:** 10.1038/s41396-022-01238-3

**Published:** 2022-04-20

**Authors:** Lucas Schmitz, Zhichun Yan, Martinus Schneijderberg, Martijn de Roij, Rick Pijnenburg, Qi Zheng, Carolien Franken, Annemarie Dechesne, Luisa M. Trindade, Robin van Velzen, Ton Bisseling, Rene Geurts, Xu Cheng

**Affiliations:** 1 Laboratory of Molecular Biology, Cluster of Plant Developmental Biology, Plant Sciences Group, Wageningen University, Droevendaalsesteeg 1, 6708PB Wageningen, The Netherlands; 2 Laboratory of Plant Breeding, Plant Sciences Group, Wageningen University & Research, Droevendaalsesteeg 1, 6708 PB Wageningen, The Netherlands; 3 Biosystematics, Plant Sciences Group, Wageningen University & Research, Droevendaalsesteeg 1, 6708 PB Wageningen, The Netherlands; 4 Shenzhen Branch, Guangdong Laboratory for Lingnan Modern Agriculture, Genome Analysis Laboratory of the Ministry of Agriculture, Agricultural Genomics Institute at Shenzhen, Chinese Academy of Agricultural Sciences, Shenzhen, China

**Keywords:** Microbiome, Plant sciences

## Abstract

The root bacterial microbiome is important for the general health of the plant. Additionally, it can enhance tolerance to abiotic stresses, exemplified by plant species found in extreme ecological niches like deserts. These complex microbe-plant interactions can be simplified by constructing synthetic bacterial communities or SynComs from the root microbiome. Furthermore, SynComs can be applied as biocontrol agents to protect crops against abiotic stresses such as high salinity. However, there is little knowledge on the design of a SynCom that offers a consistent protection against salt stress for plants growing in a natural and, therefore, non-sterile soil which is more realistic to an agricultural setting. Here we show that a SynCom of five bacterial strains, originating from the root of the desert plant *Indigofera argentea*, protected tomato plants growing in a non-sterile substrate against a high salt stress. This phenotype correlated with the differential expression of salt stress related genes and ion accumulation in tomato. Quantification of the SynCom strains indicated a low penetrance into the natural soil used as the non-sterile substrate. Our results demonstrate how a desert microbiome could be engineered into a simplified SynCom that protected tomato plants growing in a natural soil against an abiotic stress.

## Introduction

Plants sustain microorganisms around and inside their roots [1]. These communities of root-associated microorganisms are referred to as the root microbiome. There is increasing evidence showing that the root microbiome is vital to plant health, growth and development and plays a prominent role in plant fitness under diverse environmental growth conditions [2]. The root microbiome can promote growth and development by modulating plant hormone homeostasis, promoting nutrient acquisition, or improving resilience to abiotic stresses [3, 4].

The microbes that make up the root microbiome can sometimes succeed where other methods such as gene engineering have failed. For example, the HIGH-AFFINITY K^+^ TRANSPORTER 1;1 (HKT1;1) is proposed to facilitate the shoot-to-root recirculation of Na^+^, but both loss-of-function and overexpression in *Arabidopsis thaliana* (arabidopsis) does not improve salt tolerance [5, 6]. Interestingly, a strain of the soil bacterium *Bacillus subtilis* did confer salt tolerance by concurrently down- and upregulating the expression of *AtHKT1* in the roots and shoots of arabidopsis, respectively [7]. This finding of tissue-specific regulation of *AtHKT1* mediated by a microbe being critical to Na^+^ homeostasis in salt-stressed plants demonstrates the potential of plant-microbe interactions.

It is generally hypothesized that the root microbiome is also important in the case of desert plants to cope with multiple and critical threats such as nutrient deficiency, drought and salinity [8]. So far, only a limited number of studies have been conducted to characterize microbial communities associated with desert plants and their contribution to plant fitness. *Indigofera argentea* (indigofera) is a legume species that can be found in multiple desert regions [9]. It is a perennial subshrub that grows as pioneer vegetation in scattered populations in well-drained and sandy soils. Also, indigofera has a certain resilience to salt stress and can grow like a weed on former agricultural fields that suffer from high salinity due to extensive irrigation practices. For example, former agricultural areas in the Jizan desert, Saudi Arabia, are scarcely populated with indigofera (Fig. S1). We questioned whether the microbiome present in this Jizan soil plays a pivotal role in conferring abiotic stress tolerance to indigofera growing under such conditions.

Indigofera root bacterial strains of Jizan origin have been isolated and resulted among others in the identification of *Pseudomonas argentinensis* SA190, *Acinetobacter radioresistens* SA188, *Enterobacter* sp. SA187, and *Ochrobactrum intermedium* SA148. For these bacteria, plant-growth promoting effects have been predicted and experimentally verified for *Enterobacter* sp. SA187 [10–13].

Most studies focus on single strains applied to plants grown in essentially sterile conditions. This is not the case in a field setting where the presence of the local microbiome naturally implies a non-sterile environment. This non-sterile environment is suspected to explain the failure of a single strain in the field due to the competition with the local microbiome [14, 15]. Therefore, instead of this “one-microbe-at-a-time” approach [16], an alternative would be to create so-called synthetic microbial communities (SynComs), which as a community stands a better chance to survive and function in a non-sterile environment. However, it remains elusive to what extend a SynCom derived from a natural microbiome is effective in improving plant growth in a non-sterile environment, especially with the inclusion of an abiotic stress such as high salinity. And an efficient methodology of constructing and simplifying a functional SynCom is also unclear.

We characterized the bacterial microbiome of indigofera grown in Jizan soil under mimicked desert conditions and isolated strains representing the core root bacterial microbiome. Growth promoting effects of single strains and SynComs were studied on indigofera as well as the non-related crop tomato (*Solanum lycopersicum*). A SynCom of five bacterial strains promoted tomato growth under saline and non-sterile conditions. This increased salt tolerance was associated with both the differential expression of salt stress-related genes and ion accumulation in the shoot. Subsequent quantification of the relative abundance of SynCom strains revealed a low penetrance of the added SynCom, indicating that growth promotion can be triggered without affecting a native root microbiome.

## Results

### Growth promotion of indigofera by the microbiome in the Jizan soil

**Fig. 1:**
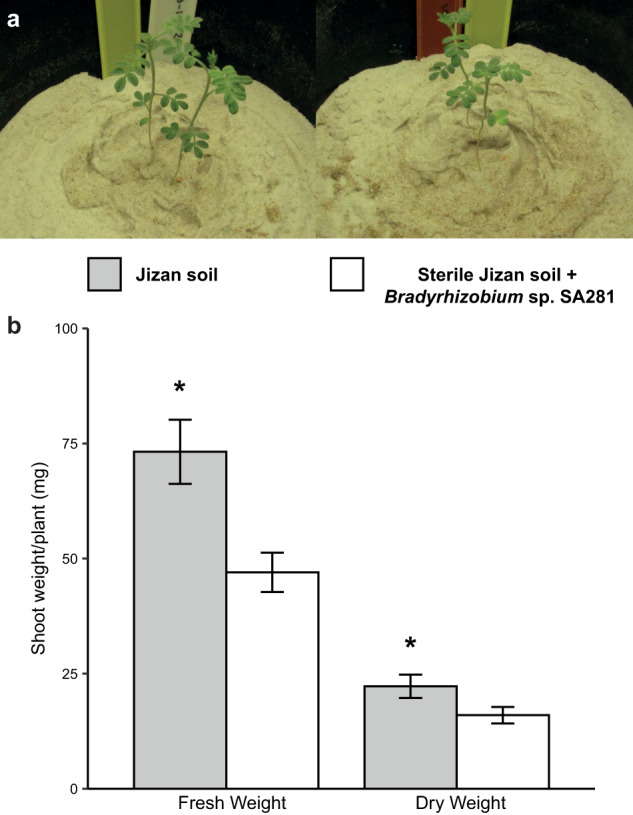
Jizan soil microbiome promotes growth of *Indigofera argentea*. **a** Six-week-old indigofera plants in Jizan soil (left) or sterile Jizan soil supplemented with the strain *Bradyrhizobium* sp. SA281 (right). There were four pots per treatment and each pot contained two indigofera seedlings. *Bradyrhizobium* sp. SA281 was isolated from nitrogen-fixing indigofera nodules grown in Jizan soil. **b** Fresh and dry shoot weight of indigofera plants at six weeks old show increased growth promotion triggered by the Jizan soil, when compared to sterile Jizan soil complemented with *Bradyrhizobium* sp. SA281. Asterisks indicate statistical significance (*p* < 0.05) as per one-way ANOVA.

To find support for the importance of the root microbiome to plant fitness, we established an assay to study the growth of indigofera mimicking native growing conditions. Indigofera did not survive when grown in sterilized Jizan soil, suggesting that the soil microbiome is essential for plant growth. Since indigofera is a legume that, under native conditions, relies on nitrogen-fixing nodule symbiosis, we repeated the growth assay now adding a compatible rhizobium microsymbiont isolated from the Jizan soil sample (strain *Bradyrhizobium* sp. SA281). This rescued plant growth and could therefore serve as an axenic control. Next, we compared plant growth in sterilized Jizan soil complemented with *Bradyrhizobium* sp. SA281 and non-sterile Jizan soil. This revealed the plants in non-sterile soil produced more biomass compared to the control condition (Fig. 1), suggesting that the native root microbiome is conducive to the growth of indigofera.

### The bacterial root microbiome of indigofera is relatively simple but distinct

**Fig. 2:**
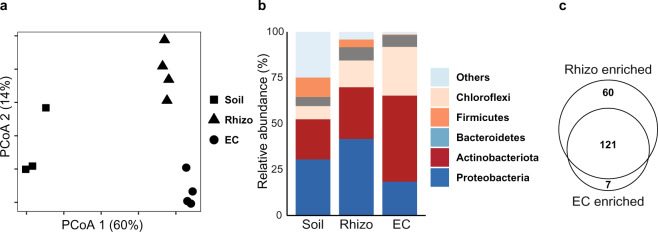
*Indigofera argentea* root microbiome in Jizan soil. **a** Principal coordinates analysis (PCoA) of the bacterial microbiomes in the soil, rhizosphere (Rhizo) and endophytic compartment (EC) of indigofera grown in Jizan soil for six weeks. The Soil is distinct from the Rhizo and EC along the first principal component. While the second component separates the Rhizo and EC. Bray-Curtis dissimilarities were calculated with OTU read counts after rarefaction and standard filtering. **b** Taxonomic classification of bacterial OTUs with relative abundance greater or equal to 5% grouped at the phylum level. The three bars represent the relative abundance of bacterial phyla in Soil, Rhizo and EC. There is a drop in bacterial diversity or number of rare phyla (indicated as “others”) from the Soil to EC. **c** Venn diagram showing number of enriched OTUs in the Rhizo and EC of indigofera.

We questioned whether indigofera recruits specific bacteria. To study its root microbiome composition, indigofera was grown in Jizan soil under mimicked native conditions. Samples from the soil, rhizosphere (Rhizo) and endophytic compartment (EC) of 42-days-old indigofera plants were collected in at least three biological replicates from which DNA was extracted. An OTU table was constructed from the Illumina sequencing reads of the 16 S rRNA gene V4 regions in these samples. Using the Bray-Curtis dissimilarity measure on the rarefied OTU table, the Soil, Rhizo, and EC samples were plotted with Principal Coordinate Analysis (PCoA) in two-dimensional space (Fig. 2a). The first two principal coordinates explained 60% and 14% of the total variance, respectively. Rhizo and EC bacterial microbiomes hardly separated along the first coordinate but were clearly distinct from the Soil community. Conversely, the Rhizo and EC samples did form separate clusters along the second coordinate. This revealed that indigofera grown in Jizan soil possessed a distinct root microbiome when compared to the soil (PCoA1 60%) and with different bacterial communities in the rhizosphere and endophytic compartment (PCoA2 14%).

In line with the PCoA, the bacterial communities in each compartment also differed at the phylum level (Fig. 2b). In the transition from the Soil to the EC, the biodiversity reduced due to a drop in the number of rare phyla (indicated as “Others”). Firmicutes and Proteobacteria were significantly depleted in the two root compartments compared to Soil, whereas the phyla of Chloroflexi and Actinobacteriota were significantly enriched in these compartments. Next, we determined the number of OTUs from the Soil that increased in their relative abundance in the Rhizo and EC. Compared to the Soil, 181 and 128 were enriched (*p* < 0.001) in the root compartments, of which 121 OTUs were shared between the Rhizo and EC (Fig. 2c & Dataset S1). Also, a similar number of OTUs was depleted in the Rhizo and EC compartments when compared to the Soil (Dataset S1 & Fig. S2). This suggests a strong rhizosphere effect of indigofera, even though the Jizan soil microbiome is relatively simple. Furthermore, there is strong commonality of bacterial community selection between the rhizosphere and endophytic compartment.

### Plant growth promotion triggered by a Jizan SynCom in indigofera and tomato

**Fig. 3:**
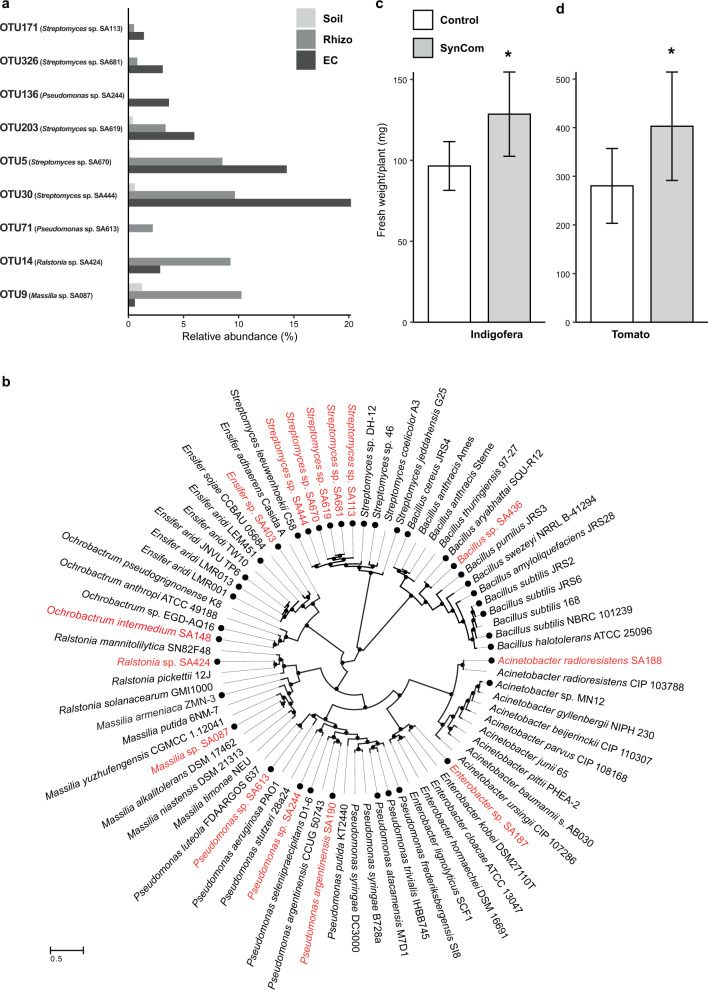
Relative abundance, phylogeny and plant growth promotion of the Jizan strains isolated from *Indigofera argentea*. **a** Jizan strains belonged to highly abundant OTUs in either the Rhizo or EC of indigofera. The *x* axis shows the relative abundance of the best matching OTU for each Jizan strain. **b** Maximum likelihood tree based on the AMPHORA gene alignments of 15 Jizan strains - colored in red. The black filled circles on the outer perimeter indicate desert-associated strains. Representative strains for each genus were also included. The distance scale indicates the number of differences between sequences. **c**, **d** Quantitative analysis of shoot fresh weight of seven-week-old indigofera (**c**) and three-week-old tomato (**d**) plants grown under sterile conditions with or without the Jizan SynCom. In both cases, the Jizan Syncom led to a better plant yield compared to the control. Asterisks indicate statistical significance (*p* < 0.05) as per Dunnett’s post-hoc test.

To study the function of the root bacterial microbiome, we aimed to isolate the bacterial strains that showed an increased abundance in the indigofera root microbiome as determined by the OTU data described above. By applying a culture-dependent approach using different cultivation media, roughly two thousand bacterial isolates were obtained. All isolates were grouped by morphology and designated with the prefix SA (Saudi Arabia) followed by a strain number. Sanger sequencing of the full length 16 S rRNA gene amplicon provided the V4 region of each isolate which could then be mapped back to an OTU found on the roots of indigofera. Ultimately, representative strains for nine of the most abundant OTUs shared between the Rhizo and EC could be identified. According to their relative abundance, these OTUs (Fig. 3a) represented approximately 40% and 30% of the Rhizo and EC, respectively, and were collectively considered the core bacterial root microbiome of indigofera. We also included the isolates *Ensifer* sp. SA403 and *Bacillus* sp. SA436, which showed promise in promoting plant growth (Fig. S3) even though they belonged to the less abundant OTUs 38 and 49 – respectively. Additionally, previous work of culture-dependent isolation on a different sample batch but from the same ecological niche culminated in four growth promoting isolates that were also included in this study: *Pseudomonas argentinensis* SA190, *Acinetobacter radioresistens* SA188, *Enterobacter* sp. SA187 and *Ochrobactrum intermedium* SA148. [10, 12, 13, 17]. The V4 sequences of the first three could be found in the indigofera microbiome (matching the OTUs 1333, 17 and 955 – respectively) though they did not belong to a dominant OTU.

Together, a total collection of 15 strains was used in further studies. It included species of the genera *Acinetobacter* and *Streptomyces* (phylum Actinobacteriota), *Bacillus* (phylum Firmicutes), and *Ensifer, Enterobacter, Massilia*, *Ochrobactrum*, *Pseudomonas*, and *Ralstonia* (phylum Proteobacteria) (Table 1). Draft genome sequencing was conducted to further characterize the selected strains (Table S1). Subsequently, maximum likelihood phylogeny was inferred from the nucleotide alignment of the 31 AMPHORA genes. This consisted of two separate analyses. First, the nine genera covering the 15 selected Jizan strains were analysed separately with reference and other root-associated strains (Dataset S2). This showed that several isolated Jizan strains are close relatives of species with reported plant-growth promoting effects. For example, *Bacillus* sp. SA436 is a close relative of the plant-growth promoting species *Bascillus megaterium* and *Bascillus aryabhattai* [18, 19], *Ensifer* sp. SA403 to the nitrogen-fixing legume symbionts *Ensifer sojae* and *Ensifer alkalisoli*, and the five *Streptomyces* sp. to *Streptomyces leeuwenhoekii*, a species known to produce a variety of specialised metabolites [20] (Fig. S4). Next, the 15 selected Jizan strains were compared to 61 earlier studied desert bacterial species of the same genera (Dataset S3). This revealed that the Jizan strains clustered together with the selected desert microbes at the genus level but diverged at the species or strain level (Fig. 3b). Taken together, this shows that the selected Jizan strains are novel yet representative of species found in desert environments.

**Table 1:** Selected bacterial strains of the root microbiome of Indigofera argentea grown in Jizan soil.

Source	Strain Code	OTU	Class	Order	Family	Genus
Current work	Highly abundant					
	SA670	5	*Actinobacteria*	*Actinobacteria*	*Streptomycetaceae*	unclassified
	SA087	9	*Beta-Proteobacteria*	*Burkholderiales*	*Oxalobacteraceae*	*Massilia*
	SA424	14	*Beta-Proteobacteria*	*Burkholderiales*	*Burkholderiaceae*	*Ralstonia*
	SA444	30	*Actinobacteria*	*Actinobacteria*	*Streptomycetaceae*	*Streptomyces*
	SA613	71	*Gamma-Proteobacteria*	*Pseudomonadales*	*Pseudomonadaceae*	*Pseudomonas*
	SA244	136	*Gamma-Proteobacteria*	*Pseudomonadales*	*Pseudomonadaceae*	*Pseudomonas*
	SA619	203	*Actinobacteria*	*Actinobacteria*	*Streptomycetaceae*	*Streptomyces*
	SA681	326	*Actinobacteria*	*Actinobacteria*	*Streptomycetaceae*	unclassified
	SA113	171	Actinobacteria	Actinobacteria	Actinomycetaceae	unclassified
	Potential PGPRs					
	SA403	38	*Alpha-Proteobacteria*	*Rhizobiales*	*Rhizobiaceae*	*Ensifer*
	SA436	49	*Firmicutes*	*Bacillales*	*Bacillaceae*	*Bacillus*
Previous work	SA148	-	*Alpha-Proteobacteria*	*Rhizobiales*	*Brucellaceae*	*Ochrobactrum*
	SA187	955	*Gamma-Proteobacteria*	*Enterobacterales*	*Enterobacteriaceae*	*Enterobacter*
	SA188	17	*Gamma-Proteobacteria*	*Pseudomonadales*	*Moraxellaceae*	*Acinetobacter*
	SA190	1333	*Gamma-Proteobacteria*	*Pseudomonadales*	*Pseudomonadaceae*	*Pseudomonas*

Eleven strains were isolated in this study, including nine strains belonging to highly abundant OTUs representing ~40% and ~30% of rhizosphere and endophytic compartment, respectively, and two low abundant or rare strains with potential PGPR traits. Four additional strains with PGPR straits were collected from previous studies. These 15 strains together were used for SynCom construction.

We questioned whether these isolated Jizan strains as a community triggered a similar plant growth promotion previously observed with indigofera grown in Jizan soil. Therefore, plants were grown in sterilized river sand that was inoculated with *Bradyrhizobium* sp. SA281. Half of the plants were also inoculated with an equal mixture of the 15 selected strains (the Jizan SynCom). Indigofera inoculated with the Jizan SynCom produced significantly more shoot biomass (42 days post-inoculation), when compared to the plants that were only inoculated with *Bradyrhizobium* sp. SA281 (Fig. 3c). This showed that the Jizan SynCom triggered increased plant growth promotion when compared to only a diazotrophic and nodulating *Bradyrhizobium* strain. Next, we questioned whether this growth response was specific to indigofera or more generic, which would be of more practical significance in translating these results to agriculture. We tested the growth response of the Jizan SynCom on tomato (Moneymaker cultivar). The Jizan SynCom also triggered a significant growth response in tomato (Fig. 3d). This demonstrated that the growth-promoting effect of the bacterial Jizan strains is a generic effect on plants.

### Jizan SynCom promotes tomato growth under salt stress and non-sterile conditions

We questioned whether the Jizan SynCom is also effective in promoting abiotic stress tolerance in other plant species and under non-sterile conditions. To test this, we used tomato and established a generic assay for analysing microbial effects on plant fitness under various conditions with the focus on salt stress in this study (Fig. S5). A controlled and reproducible non-sterile substrate was created by mixing sterilized river sand with 10% of a well characterized soil and supplemented with nutrient solution. We wanted to exclude interference from the endogenous SynCom strains present in the soil and evaluate SynCom effectiveness in the presence of another natural microbial community outside its native habitat. So, we collected soil from an ecological field station (the Mossel area at Veluwe, the Netherlands), referred to as the Mossel soil, of which the rhizosphere effect was characterized on a series of plant species [21]. Diluting the soil with sand allowed us to design a synchronized growth assay in which the effects of soil nutrients were reduced. The physical properties of the sand were also more suitable for the salt stress assay. Tomato seeds were sown in the 10% soil - 90% sand mixture and inoculated with/without the Jizan SynCom (approximately 10^9^ cells per plant). After one week of growth in this soil mixture, plants were exposed to various salt levels (0, 100, 200 and 300 mM NaCl) (Fig. S5). Two weeks post salt imposition, the total plant biomass was quantified. First, we noted that under non-sterile conditions the Jizan SynCom promoted tomato growth in the absence of salt (Fig. S6). Furthermore, the biomass was not significantly different between control and SynCom treated plants except for those exposed to 200 mM NaCl. In contrast, tomato plants without Jizan SynCom inoculation showed a clear decline in biomass proportional to the salt concentration. These results showed that the Jizan SynCom not only promoted tomato growth but also conferred tolerance to salt imposition. Since the 200 mM salt level provided a clear contrast in plant growth between the Jizan SynCom inoculated plants and non-inoculated control plants, it was set as the standard salt concentration for subsequent experiments in this study.

### Bacterial SynCom triggered salt stress resilience associates with differential expression of salt stress related marker genes and ion content accumulation

**Fig. 4:**
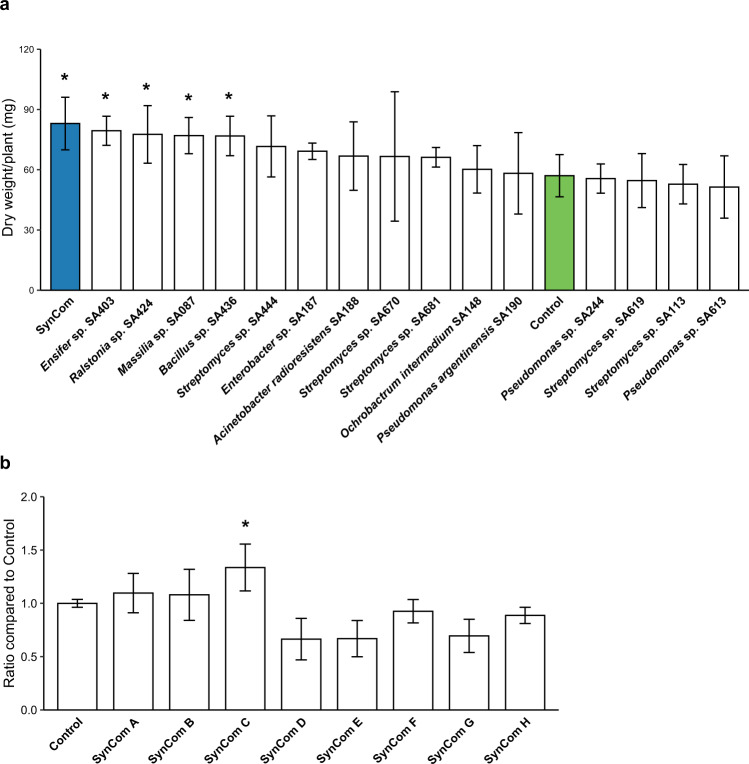
Growth promotion of the SynComs and individual Jizan strains on salt-stressed tomato plants. **a** The dry shoot weight of three-week-old tomato plants grown in the non-sterile substrate and inoculated with either a single Jizan strain or the SynCom as an equimolar mixture. Salt imposition was with 200 mM NaCl according to the assay described previously (Fig. S4). The SynCom (colored in blue) performed best and together with the strains *Ensifer* sp. SA403, *Ralstonia* sp. SA424, *Massilia* sp. SA087, *Bacillus* sp. SA436 were significantly different from the control plants (colored in green) which were not inoculated. **b** Ratios of the shoot dry weight of tomato plants treated with simplified SynComs to the control. Each SynCom contained four or five Jizan strains and the control was not inoculated. The five member SynCom C performed the best and was the only one significantly different to the control. Asterisk indicates statistical significance (*p* < 0.05) as per Dunnett’s test.

We questioned whether a simplified Jizan SynCom can trigger salt tolerance under non-sterile conditions. First, individual strains were tested. Tomato plants were grown as described above, inoculated with 15 strains individually and exposed to 200 mM NaCl. Plants growing only in the non-sterile substrate served as an inoculum-free control. Of the 15 strains tested, *Ensifer* sp. SA403, *Ralstonia* sp. SA424, *Massilia* sp. SA087, and *Bacillus* sp. SA436 promoted tomato growth compared to the non-inoculated plants. The remaining strains did not significantly affect plant growth when compared to the control plants (Fig. 4a). Interestingly, the Jizan SynCom inoculated plants showed the highest shoot biomass compared to other inoculated plants.

Next, we tested simplified SynComs containing a subset of the 15 strains from the Jizan core microbiome. Instead of using a targeted approach, we decided to randomly combine the strains. We did avoid taxonomic redundancy so that none of the simplified SynComs would have two or more strains of the same genus. In total, 20 combinations of 3 to 5 strains were tested on tomato plants growing in the non-sterile substrate and exposed to 200 mM NaCl (Table S2). This revealed that SynCom C, which consisted of *Massilia* sp. SA087, *Enterobacter* sp. SA187, *Ensifer* sp. SA403, *Bacillus* sp. SA436, and *Streptomyces* sp. SA444 led to the strongest growth response, having a 34% increase in dry shoot biomass compared to the non-inoculated control (Fig. 4b) and even outperforming the 15 strain Jizan SynCom.

**Fig. 5:**
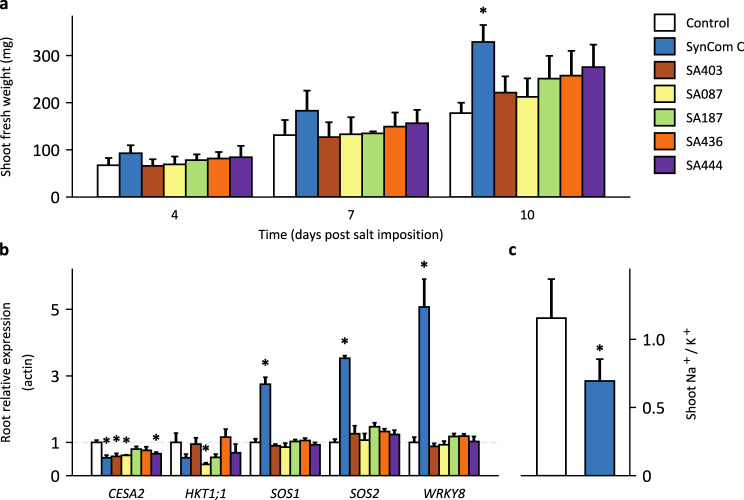
SynCom C induces salt stress tolerance related responses in tomato. Tomato seeds were sown in sterilized river sand and inoculated with the five-member SynCom C or the five individual strains. Plants were exposed to 200 mM NaCl on day seven. The root and shoot tissue were harvested on four, seven, and ten days post salt imposition. **a** Shoot fresh weight of the tomato plants for the three time points after salt imposition. A linear model was fitted to the biomass with the time points and inoculation as the explanatory variables. Only the SynCom C treated samples had a significantly different (*p* < 0.05) slope from the inoculum-free control samples. **b** Relative expression of salt stress resilience-related genes to Actin in the root tissue of tomato plants four days post salt imposition. An asterisk indicates a significant (*p* < 0.05) difference from the control, according to Dunnett’s test. **c** The Na^+^/K^+^ ratio in the shoot of tomato plants ten days post salt imposition. SynCom C inoculated plants had a significantly lower ratio than the control (tested with Student’s *t* test). SynCom C is composed of the strains *Massilia* sp. SA087, *Enterobacter* sp. SA187, *Ensifer* sp. SA403, *Bacillus* sp. SA436 and *Streptomyces* sp. SA444.

We questioned whether the increased biomass of salt-treated tomato plants inoculated with SynCom C is the result of a generic growth response, or alternatively, associated with salt stress-related physiological factors such as ion homeostasis or the expression of salt stress-related marker genes. To this end, leaf and root tissues were sampled from tomato at four, seven and ten days post salt imposition. Plants were grown either sterile (control), inoculated with SynCom C or with the five individual strains which compose this best performing SynCom. By including the five strains as separate inoculums, we aimed to validate that simultaneous presence of different strains as a SynCom is a prerequisite for the observed growth response. Quantification of the fresh shoot biomass showed that SynCom C was the only inoculum that significantly promoted tomato growth compared to the inoculum-free control (Fig. 5a). Transcriptional analysis was then performed on root and shoot tissue for the salt stress related marker genes *CELLULOSE SYNTHASE A2* (*CESA2*), *HKT1;1, SALT OVERLY SENSITIVE 1* (*SOS1*), *SOS2* and *WRKY8* (Table S3). Four days post salt *imposition, SOS1, SOS2* and *WRKY8* expression was significantly upregulated in the root of tomato plants treated with SynCom C compared to the non-inoculated control (Fig. 5b). This effect was not observed in the roots of plants inoculated with the individual strains. Conversely, in the shoot there was a significant downregulation of *SOS2* for the individual strains but not SynCom C (Fig. S7f). Interestingly, *HKT1;1* was upregulated in the shoot by SynCom C and three of the individual strains though none were significantly different to the control. This stands in contrast to the expression of the same gene in the root tissue where *HKT1;1* is downregulated by four of the inoculums including SynCom C. The ion content of the shoot tissue from the control and SynCom C inoculated plants was measured at ten days post salt imposition. This revealed that the Na^+^/K^+^ ratio was significantly lower in SynCom C inoculated plants, when compared to control plants (Fig. 5c). To determine whether this effect is also observed in presence of a soil microbiome, the experiment was repeated but now plants were grown in the 10% Mossel soil + 90% sand mixture. Again, this showed the growth promoting effect of SynCom C under saline conditions and with a significantly lower Na^+^/K^+^ ratio than the control plants (Fig. S8b & d). Taken together, these results demonstrate that SynCom C triggers increased resistance to salt stress in tomato plants grown in non-sterile conditions.

### The Jizan SynCom colonized the root of tomato plants under non-sterile conditions

The degree of root colonization due to some treatment as an indicator of strain importance is the dominant approach taken in microbiome studies. This strategy also lends itself to the study of the inter-bacterial dynamics in either a native microbiome or, in our case, the interaction between a SynCom and its environment. As the 15 Jizan strains correspond to abundant OTUs in the root compartments of indigofera, their root colonization especially as a function of salt level could be a key factor leading to a successful plant phenotype.

To identify traits other than the growth promotion of single strains that can be used in the design of a SynCom, we conducted an experiment to measure the root colonization of the 15 Jizan strains in a non-sterile environment. Tomato plants were grown in the non-sterile substrate and half were inoculated with the Jizan SynCom. Plants were exposed to a single salt concentration ranging from 0 to 300 mM NaCl in 100 mM increments. The V4 16 S rRNA gene region was sequenced with the Illumina Hiseq2500 platform on DNA isolated from the Soil, Rhizo and EC of each sample. Amplicon sequence variants (ASVs) were inferred from the sequencing reads and, after standard filtering, resulted in 10,029,248 reads distributed over 3766 measurable ASVs.

**Fig. 6:**
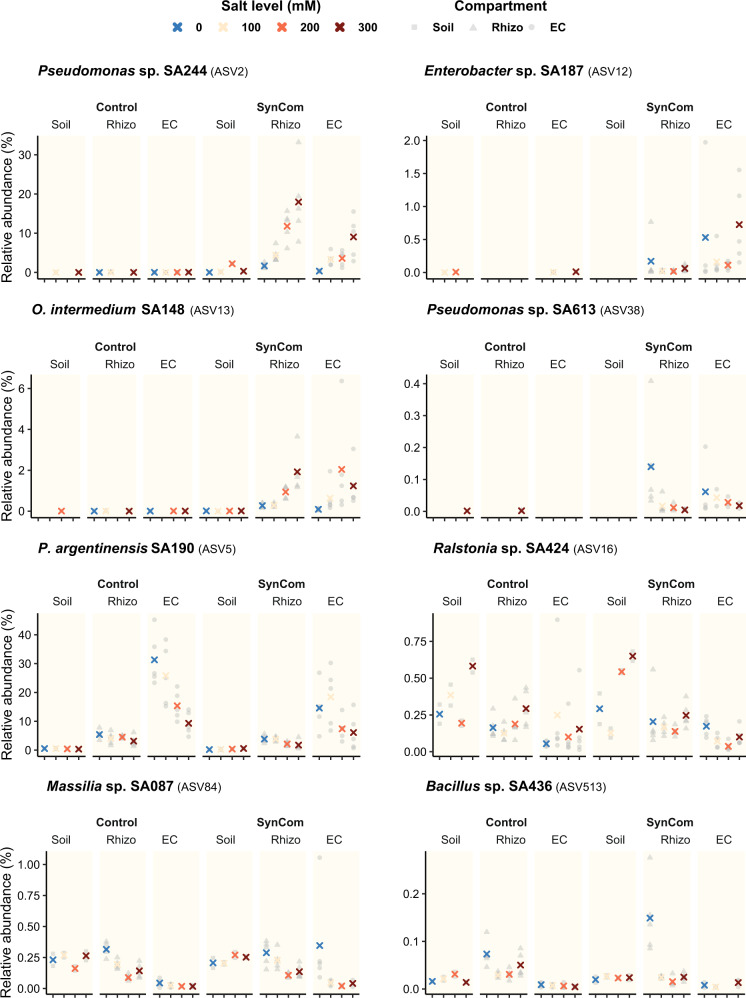
SynCom colonization at different salt levels in the soil and roots of tomato. Relative abundances of the bacterial ASVs in the Soil, Rhizo and EC of three-week-old tomato plants inoculated with or without the Jizan SynCom. Plants were grown in the non-sterile substrate and were exposed to a salt gradient. The eight ASVs with a perfect match to the V4 region of the Jizan strains are shown here. The other strains fell below our filtering criteria (Fig. S8). The relative abundances of the replicates are shaded as gray symbols per compartment and the mean is shown as a cross with the color referring to the salt level.

With the V4 subregion from the 16 S rRNA gene in the genome assemblies assumed as the expected sequence, the Jizan strains could be matched to ASVs which served as an indicator for strain presence and relative abundance (Table S4). This analysis showed that of the 15 Jizan strains, the ASVs of 8 strains were found among the measurable ASVs (designated as targeted ASVs). While the other seven strains, including two members of SynCom C (*Ensifer* sp. SA403 and *Streptomyces* sp. SA444), are not shown because they fell below the filtering criteria (Fig. S9). The relative abundances of each targeted ASVs in the three compartments were plotted along the salt gradient (Fig. 6). Out of the eight targeted ASVs, ASV2 (*Pseudomonas* sp. SA244), ASV12 (*Enterobacter* sp. SA187), ASV13 (*O. intermedium* SA148) and ASV38 (*Pseudomonas* sp. SA613) as well as ASV91 (*Ensifer* sp. SA403) were not detected in the control but only in the soil inoculated with the Jizan SynCom. This indicated that the bacteria with these ASVs were likely not present in the non-sterile substrate and probably originated from the Jizan strains. Apart from the four targeted ASVs that were absent, ASV5 (*P. argentinensis* SA190), ASV16 (*Ralstonia* sp. SA424), ASV84 (*Massilia* sp. SA087), ASV513 (*Bacillus* sp. SA436) were detected both in the control and SynCom-inoculated samples. This prevents the differentiation of these four Jizan strains from the endogenous microbes present in the non-sterile substrate. Linear regression analysis showed that ASV2 (*Pseudomonas* sp. SA244) and ASV13 (*O. intermedium* SA148) significantly correlated with the salt level in the EC and, in the case of ASV2, also in the Rhizo (Fig. S10), which suggested the two strains belonging to these targeted ASVs were able to successfully colonize the roots under increasing saline conditions.

**Fig. 7:**
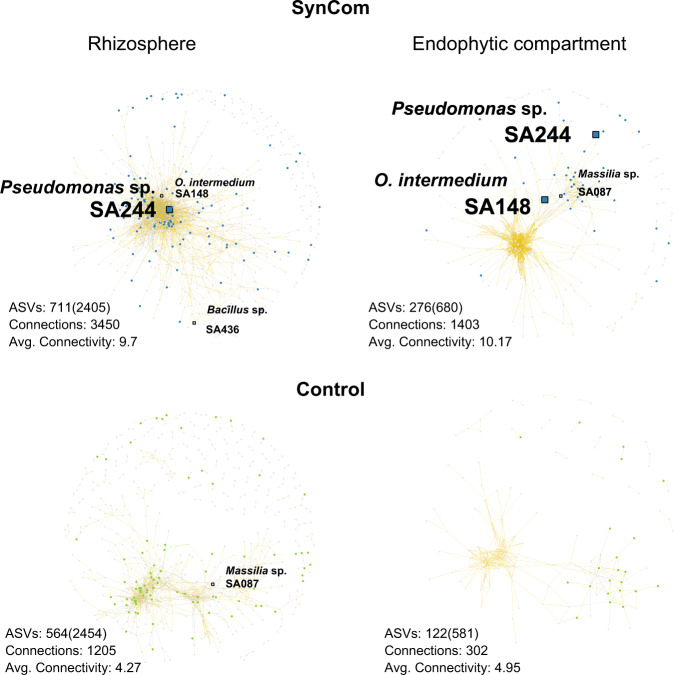
Bacterial co-occurrence networks in the root of tomato across a salt gradient. Networks are based on Spearman’s rank correlation between the ASVs in the Rhizo and EC of tomato growing in the non-sterile substrate with or without SynCom across a salt gradient from 0 to 300 mM NaCl. Only edges with a correlation score |ρ | > 0.7 and a *p* < 0.001 are shown. Positive and negative correlations are colored as gold and gray lines, respectively. Each node in a network is a detected ASV which means it passed these selection criteria. The square nodes are the ASVs belonging to a Jizan strain from the SynCom. A blue or green for the SynCom and control, respectively, indicates an ASV that was significantly enriched in any of the salt levels. The Jizan strains which were detected as well as enriched in a salt level are enlarged for clarity. Additional properties are shown below each network; ASVs: number of detected ASVs (initial number of ASVs), Connections: total number of edges, Avg. Connectivity: average number of edges per node.

Moreover, bacterial network analysis was performed and the co-occurrence of targeted ASVs was determined as a function of the salt level. The bacterial co-occurrence analysis showed that ASV2 (*Pseudomonas* sp. SA244) and ASV13 (*O. intermedium* SA148) were always present in both root compartments; while ASV513 (*Bacillus* sp. SA436) and ASV84 (*Massilia* sp. SA087) were only present in the Rhizo and EC, respectively. The presence of the Jizan SynCom significantly increased the number of connections and, as a result, the average connectivity in the Rhizo and EC networks (Fig. 7). As ASV2 and ASV13 were highly abundant in the root compartments, these two strains may have contributed to changes in the microbial networks, though their functions in the bacterial networks in the root compartment are yet unknown. Taken together, the presence of the SynCom C members was confirmed in this experiment, but only two of them could be definitively distinguished from the natural microbiome already present in the non-sterile substrate.

## Discussion

In this study, we showed that a bacterial SynCom originating from the Jizan desert plant indigofera promotes growth of the important economic crop tomato under salt stress in the presence of a non-sterile substrate that mimics a natural soil microbiome. Furthermore, we simplified this SynCom from the initial 15 to 5 strains. This simplified SynCom outperformed the plant growth promoting effect observed with the initial SynCom under the same experimental conditions. The five selected strains originate from the roots of the desert plant indigofera grown in a native soil. We argue that crop cultivation under abiotic stresses can be improved with microbiomes from such environments.

Some studies took a similar approach exploring desert microbiomes for the discovery of plant growth promoting rhizobacteria. For example, microbial communities have been analyzed from different desert regions, like Jizan, Thar, Atacama, Kalahari, Namib and Sahara deserts, as well as from various desert plant species [3, 22–27]. Bacterial strains belonging to the genera *Pseudomonas, Bacillus, Klebsiella, Cupriavidus, Ochrobactrum, Isoptericola*, and *Enterobacter* were isolated and showed to promote growth of multiple plant species under saline conditions, including model plant species arabidopsis as well as crops like wheat (*Triticum aestivum*), rice (*Oryza sativa*), alfalfa (*Medicago sativa*) and tomato [28, 29].

In our opinion, confirming the plant growth promoting response of rhizobacteria is a critical step in determining the robustness of such a trait. Plant growth promoting responses under both abiotic and biotic stress induced by single strain inoculants has been well documented. Unfortunately, these strains often fail when they are applied individually in the field, which is attributed to competition by the local microbiome [30]. For this reason, there has been a shift from this so-called “one-microbe-at-a-time” approach to creating synthetic microbial communities [16]. The general idea is that a community of microbes will be more competitive by forming a stable community and will be able to maintain the traits beneficial to plant growth in the field [31]. Other studies have attempted to combat salt stress with SynComs albeit in sterile conditions [32–34]. However, to our knowledge there are no studies where a SynCom provides protection against salt stress to plants grown in the presence of a natural or local microbiome as seen under field conditions.

The quantification of shoot biomass was considered as the indicator for SynCom performance. From this data, it is unclear if the five member SynCom (SynCom C) leads to a higher biomass due to a specific microbe-mediated salt stress response or a general plant growth promotion. To verify, we measured the transcription of a selected list of salt stress related genes in tomato after SynCom C inoculation with a salt stress. This revealed that four days post salt imposition, the SynCom C mediated an early salt stress response in both the root and shoot tissues of tomato. For example, *SOS1, SOS2* and *WRKY8* were significantly upregulated in the root four days post salt imposition. The most common causes of salt stress on plant growth are ion toxicity [35], osmotic stress [36], and the accumulation of reactive oxygen species (ROS) [37]. As salt imposition causes large influxes of Na^+^ into plant tissue, a critical step in the plant response is to recirculate and sequestrate Na^+^, as well as prevent further influx via the root [38]. The well-studied salt overly sensitive (SOS) pathway plays a crucial step in preventing Na^+^ accumulation [39]. SOS3 is a calcium binding protein that senses cytosolic Ca^2+^ changes, caused by salt stress, and in turn interacts with SOS2, which belongs to the SnRK3/calcineurin-interacting protein kinase (CIPK) subfamily. The SOS3/SOS2 kinase complex phosphorylates the Na^+^/H^+^ exchanger SOS1, which is fundamental in Na^+^ extrusion, distribution and long-distance transport in tomato [40]. Mutant studies of *SOS1* show that it is essential for tomato NaCl tolerance, as gene silencing results in reduced growth [41]. It is therefore interesting that we find an upregulation of *SOS1* in SynCom C inoculated plants in the presence of salt. Likewise, *SOS2* is highly upregulated four days post salt imposition, indicating that the SOS pathway is more active in SynCom C treated tomato plants than in the non-inoculated control. In addition, *HKT1-like* transporters are also essential in Na^+^ recirculation and extrusion [42]. In arabidopsis and rice, ectopic expression of *HKT1;1* can increase Na^+^ exclusion from the shoot [43, 44]. While the effect was not significant, there was an upregulation by SynCom C of *HKT1;1* in the shoot of tomato plants four days after the salt imposition [45]. These findings suggest that SynCom C plays a role in the avoidance of ion toxicity, prompting an early activation of plant ion homeostasis mechanisms. This is in line with the lower Na^+^ /K^+^ ratio in the shoot of SynCom C treated tomato plants. Additionally, none of the five SynCom C strains when applied as a separate inoculum showed a significant effect on plant biomass, nor was there a noticeable difference in the expression of genes that would indicate a salt stress response. This supports that SynCom C as a community is required for increasing salt stress resilience in tomato.

With the success of SynCom C, derived from the Jizan SynCom, we could evaluate the reported methods to design a suitable SynCom from a bacterial library. The key characteristics reported in literature rely on obvious traits such as plant growth promoting potential of single strains and, within the field of microbiome research, the presence of microbes in and around the roots [31]. A popular method to identify key species in a natural microbiome is based on the read counts of microbial sequences as an estimate of microbe presence [46]. This lends itself to the idea of a core microbiome and dominant OTUs, which we used to guide the isolation and selection of the original strains from the Jizan soil microbiome. The correlation between the abundance of a single strain and a triggered effect, such as abiotic stress tolerance, is considered to be indicative of a strain’s relative importance [47]. By extending this idea to the interactions between strains, network analysis can reveal the effect of a treatment on microbiome structure, which can further guide the identification of key species [48]. However, the key characteristics of the SynCom C strains did not meet the above expectations. Starting with the evaluation of plant growth promotion as a trait, only three of the five strains belonging to SynCom C significantly improved tomato growth under the same conditions when inoculated individually: namely *Massilia* sp. SA087, *Ensifer* sp. SA403, and *Bacillus* sp. SA436. The other two strains (*Enterobacter* sp. SA187 and *Streptomyces* sp. A444) showed a non-significant increase in biomass compared to the control. Interestingly, the community consisting of the best performing strains (SynCom A) did not significantly outperform the non-sterile control. This suggests that the selection criteria in the design of a SynCom should not solely rely on the plant growth promotion by a strain.

Shifting focus to the presence of microbes in or near the root, we would expect the five SynCom C strains to be dominant in relative abundance, positively correlated to the salt level and play a leading role in microbial network structure. Even though all SynCom C strains can be detected when added to a natural microbiome in combination with salt stress, none of the strains was dominant in abundance relative to the other strains detected from the Jizan SynCom. Interestingly, the only two strains (*Pseudomonas* sp. SA244 and *O. intermedium* SA148) that showed a positive correlation between read count and salt level did not belong to SynCom C. None of the SynCom C members showed a similar response to the salt gradient. Inoculation of the 15 strains from the Jizan SynCom influenced the microbial network of the natural microbiome by increasing the average connectivity. The network analysis revealed that only two members from SynCom C passed the detection threshold: *Bacillus* sp. SA436 and *Massilia* sp. SA087 in the rhizosphere and endophytic compartment, respectively. Both these strains were positioned in the periphery of their respective networks, which suggests a minor interaction with the natural microbiome. The SynComs containing *Pseudomonas* sp. SA244 and *O. intermedium* SA148, which were also present in the network and highly connected with the natural microbiome, did not perform as well as SynCom C. This suggests that solely relying on read count abundances from meta-amplicon sequencing is also not a reliable method for the design of a SynCom.

Overall, in this study, we isolated a core root bacterial microbiome of the desert plant indigofera and constructed a 15 strain SynCom that possesses a robust plant growth promoting effect on tomato when exposed to salt stress. The reduction of this Jizan SynCom led to a simplified version in the form of SynCom C, which retained its plant growth promoting effect. This SynCom was effective in specifically combating salt stress and did not lose effectiveness in a non-sterile environment. Our data suggests that a promising approach or strategy is in the combination of plant phenotype screening and more advanced and accurate methods, such as metagenomic, metatranscriptomics and metabolomics sequencing, to better model the predictive traits for a successful SynCom design.

## Materials and methods

### Bacterial strains and culture conditions

Bacterial strains were cultured in tryptic soy broth medium (TSB) at 28 °C. Fast growing strains were cultivated in 1/10th TSB for 1 day and slow growing strains were cultivated in ½ strength TSB for 5 days. Rhizobial strains were cultivated in yeast extract manitol (YEM) for 3–4 days. Bacterial cells were collected by centrifugation, washed three times with 0.9% NaCl and resuspended in appropriate solution according to the plant assay. The single strain cultures were adjusted to a final density of 0.5 × 10^8^ CFU ml^−1^. The SynComs were an equimolar mixture of strains with a final concentration of 5 × 10^8^ CFU ml^−1^ and each plant received in total 10^9^ cells.

### Plant assay for indigofera and tomato

Indigofera seeds were surface sterilized by washing with ethanol and soaking in ¼ bleach for 10 min. Seeds were kept at 4 °C for three days followed by a seven-day incubation at 30 °C. After germination, two seedlings were transplanted per pot containing either sterile river sand supplemented with *Bradyrhizobium* sp. SA281 or Jizan soil. The Jizan soil was collected from the Jizan desert in Saudi Arabia (Latitude 16.9405 N; Longitude 42.6119E) and has a low nutrient content (Table S5). The river sand was sterilized by gamma radiation with a minimum dose of 25 kGy which is fatal to most microbes [49]. The water holding capacity (WHC) of the substrate was maintained at 30% with sterilized dH_2_O. The climate room was configured at 35/25 °C day/night, 12 h of light and 75% relative humidity to mimic the Jizan desert condition. The second indigofera experiment with the Jizan SynCom was identical in growing conditions and seed preparation. Two seedlings were transplanted to pots with sterile river sand inoculated with the Jizan SynCom or only with *Bradyrhizobium* sp. SA281 as a control. The sample size per treatment was four with two seedlings per replicate or pot. WHC was again maintained at 30% but *Fahräeus* medium was used once a week instead of sterile dH_2_O. In both experiments, fresh and dry shoot weight were measured at day 42 post transplantation.

Tomato seeds of the MoneyMaker cultivar were sterilized with the same method. After vernalization, seeds were germinated in one day at 25 °C in the dark. The germinated seeds were sown into plastic cups containing, according to the experiment, either sterile river sand or the non-sterile substrate which was a 10/90% mixture of Mossel soil sourced from the Veluwe (the Netherlands) and sterile river sand. Inoculation by either a single Jizan strain or the 15-member Jizan SynCom followed immediately after sowing. Uniform seedlings were selected 7 days post sowing and the fresh weight recorded at day 21. Pots were randomly repositioned in the growing chamber (25 °C, 12 h lighting, 60% relative humidity) several times throughout the growing period. Where applicable, the salt imposition always occurred on day 7 according to a modified protocol from a previous study [50] (Fig. S4). Pots were first watered to 60% WHC before soaking them in a saline solution for 30 min. The inflow of the saline solution will mix with the water present in the substrate and (after returning to 60% WHC) a target salt concentration is reached. For details refer to Fig. S4.

### DNA extraction and sequencing

The soil, rhizosphere and endophytic compartment were isolated from the roots of tomato or indigofera following the procedure described previously [21]. In summary, the soil was defined as the sand easily removed from the roots by shaking. The roots were washed in sterile phosphate buffer (6.33 g NaH_2_PO_4_.H_2_O, 16.5 g Na_2_HPO_4_.7H_2_O & 200 µL Silwet L-77 in 1 L dH2O, pH = 7.0) and vortexed briefly. After removing the roots, the suspension was filtered through a 100 µm cell strainer and spun down for 10 min at 4000 x *g*. Supernatant was discarded and the remaining pellet was the rhizosphere. For the EC: the roots were washed twice more with sterile phosphate buffer, transferred to a new tube with sterile phosphate buffer, sonicated for 10 min (with a 30 s pause in every minute) and dried on sterile filter paper. All samples were frozen in liquid nitrogen and stored at −80 °C. DNA from soil and rhizosphere samples was isolated using the Mo Bio PowerSoil kit (Qiagen) according to the manufacturer’s instructions. According to the procedure described previously [51], DNA from the EC samples was isolated using the Fast DNA Spin Kit for Soil (MP Biomedicals). Quality and quantity of the DNA was checked by nanodrop and gel electrophoresis. Per sample, around 400 ng was sent for 16 S rRNA gene sequencing at Beijing Genomics Institute (BGI) with the V4 primers 515 F and 806 R. The samples from indigofera and tomato were sequenced with MiSeq and HiSeq, respectively.

### Meta amplicon sequencing data processing

For the indigofera sequencing data, paired-end FASTQ reads were merged into contigs using PANDASeq (v.2.3) with a minimum overlap of 50 bp, minimum contig length of 100 bp and a Phred score of 25. Contigs were converted to FASTA format with the fastx-toolkit (v.0.0.13) into a single file. These reads were clustered into operational taxonomic units (OTUs) according to the UPARSE pipeline [52] implemented in VSEARCH (v.1.1.3) [53]. In short, the pipeline consisted of de-replication, sorting by abundance, and discarding singletons. OTUs were generated with the UPARSE algorithm and chimeric sequences were removed with the UCHIME algorithm. An OTU table was constructed by mapping reads back to the OTUs with the “usearch_global” algorithm from VSEARCH. Taxonomy based on the SILVA (V138) [54] 16 S rRNA gene dataset was assigned to the OTUs with the RDP classifier (v.2.10.1). All processing steps were implemented in a SnakeMake workflow.

For the tomato sequencing data, amplicon sequence variants (ASVs) were inferred from the Illumina paired-end FASTQ reads with the DADA2 pipeline (v.1.12.1) [55]. FASTQ reads were filtered with the filterAndTrim function allowing for only one expected number of errors (maxEE = 1) and discarding reads with any ambiguous nucleotides (maxN = 0). Error rates were learned separately with the first 1 × 10^8^ nucleotide bases of the filtered forward and reverse reads. The pseudo-pooling algorithm from the dada function together with the learned error rates predicted the ASVs in both orientations of the filtered reads after dereplication. The paired reads were merged with the mergePairs function and chimeras were removed by the consensus method with the removeBimeraDenovo function. The RDP Naive Bayesian Classifier algorithm [56] as implemented in the assignTaxonomy function assigned the taxonomy of the ASVs against the SILVA (v138) dataset.

### Meta amplicon sequencing data analysis

All analyses were performed in the R environment (v.3.6.3). The bacterial dataset of indigofera, which was the OTU table, was processed as described previously [21]. First, OTUs related to mitochondrial and chloroplast sequences were removed and it was named as “raw OTUs”. Next, the OTUs that have more than 25 reads in any sample were kept and they were named as “measurable OTUs”. The result was a table with 873 measurable OTUs with over 728,970 sequences divided over 11 samples (Dataset S4). Using the Bray-Curtis dissimilarity measure on a rarefied measurable OTU table, the soil, Rhizo and EC samples of indigofera were plotted with principal coordinate analysis (PCoA) in two-dimensional space. This was largely done with the vegan package (v.2.5.6).

For the bacterial dataset of tomato, which was the ASV table, ASVs related to mitochondrial and chloroplast sequences were removed. Then, ASVs with sequence length smaller than 253 or bigger than 254 bps were excluded. After this, data relating to our study were subset and named as “raw ASVs”. In order to track the SynCom members in our sequencing data, the V4 region of these strains were extracted and aligned with the sequences of “raw ASVs”. The “raw ASVs” table was then filtered and ASVs (which have more than 25 reads in at least 5 samples) and named as “measurable ASVs”. The custom R commands were used in these analyses, mainly retrieved from the R packages tidyr (v.1.1.1), reshape2 (v.1.4.4), ggplot2 (v.3.3.2) and fmsb (v.0.7.0).

For differential abundance test, we used the same method as implemented in a previous study [21]. To test OTUs differently abundant between the Rhizo and EC in comparison to Soil, we used the default setting to create the normalized OTU table from “raw OTUs”. Next to it, the fitZig function and default settings in the metagenomeSeq package (v.1.28.2) [57] were used for differential abundance testing. This was the same for tomato where the “raw ASVs” were tested for differential abundance between no salt (0 mM) and each salt treatment (100, 200, 300 mM NaCl input) for all three compartments. Furthermore, for the enriched ASVs, the correlation between their abundance (log2-normalized reads) and increasing salt gradients was tested with linear regression.

We constructed co-occurrence networks between bacterial ASVs embedding all salt levels in three compartments in both control and SynCom inoculation conditions, using a custom implementation of publicly available scripts [58]. For these networks, we used the normalized ASV table and conducted Spearman rank correlations between ASVs. The networks were visualized with the Fruchterman-Reingold layout (50,000 permutations) in igraph and show the strong and significant correlations (|ρ | > 0.7 and *p* < 0.001).

### Bacteria isolation and correlation analysis

To isolate strains from the rhizosphere and endophytic compartment of indigofera grown in the Jizan soil, serial dilutions of the glycerol stocks obtained from these compartments were plated on 1/10th TSA, King’s B, R2A and ISP3 agar media. Plates were incubated at 28 °C for 14 days. Colony appearance was monitored daily and independent colonies were re-streaked on 1/10th TSA plates. Colonies were re-streaked on fresh 1/10th TSA plates once more to ensure purity. The 16 S rRNA gene was amplified with primers 63 F 5′-CAGGCCTAACACATGCAAGTC-3′ and 1389 R 5′-ACGGGCGGTGTGTACAAG-3′ [59] of fresh colonies in replicate. PCR products were sequenced at Macrogen (Amsterdam, the Netherlands). All 16 S rRNA sequences were processed with Geneious 8.1.9 (https://www.geneious.com) and submitted to RDP database for taxonomic identification. For the correlation analysis, the V4 region of the 16 S rRNA gene sequences were aligned with the consensus sequences of the indigofera OTUs. Isolates with the V4 region matching OTUs with more than 97% identity were kept. Representative isolates of these OTUs were selected for strain level analysis by using BOX-PCR with primer BOXA1R 5′-CTACGGCAAGGCGACGCTGACG-3′ [60] and repetitive strains were removed by comparing the genetic profiles. In the end, 11 strains were selected including 9 strains belonging to high abundant OTUs and 2 strains belonging to relatively low abundant OTUs but potentially promoting plant growth. Their glycerol stocks were prepared and stored at −80 °C.

### Genome assemblies and phylogeny

Illumina paired-end FASTQ files were cleaned with Trimmomatic (v.0.38) [61] by clipping reads if the average Phred quality score within four consecutive bases dropped below 28 (SLIDINGWINDOW:4:28). The trimmed FASTQ files were then assembled into contigs by SPAdes (v.3.12.0) [62] with the careful option turned on and k-mer sizes of 21, 33, 55, and 77 nt. A high amount of assembly contamination (up to 79%) was detected by CheckM (v.1.1.2) [63] in the following genera: *Bacillus* (SA436), *Ensifer* (SA403), *Massilia* (SA087), *Pseudomonas* (SA613 and SA244), *Ralstonia* (SA424), and *Streptomyces* (SA113, SA444, SA619, SA670 and SA681). These assemblies were cleaned with K-means clustering (Python Scikit-learn) [64] on the tetranucleotide signatures of the contigs. A second round of CheckM indicated a drop in contamination to below two percent for all assemblies. Finally, assembly statistics and genome completeness were assessed with QUAST (v.5.0.0) [65].

The phylogeny of the Jizan strains was inferred by maximum likelihood on concatenated AMPHORA gene alignments. For the nine genera, candidate accessions were selected from the NCBI RefSeq database based on several criteria. First, all the reference assemblies from each genus were included. Additional accessions were either plant root-associated or isolated from a desert habitat. The coding domain sequences and translated protein sequences were downloaded from NCBI in September 2020. For the Jizan strains, the open reading frames (ORFs) on the contigs were predicted by Prodigal (v.2.6.3) [66]. Hidden Markov Model profiles of the AMHPORA proteins were downloaded from the AMPHORA2 GitHub repository [67]. These were used by hmmsearch (v.3.2) [68] to identify the best protein hit in the translated ORFs with an E-value threshold below 0.001. Accessions without the full set of 31 AMPHORA genes were discarded from the analysis. The AMHPORA genes were aligned separately with Clustal Omega (v.1.2.4) [69] and inspected manually. A multiple sequence alignment (MSA) was constructed by concatenating the AMHPORA gene alignments per accession. Phylogeny was inferred on the MSA with IQTree (v.1.6.12) [70]. The affiliated tool ModelFinder [71] identified the best-fit substitution model for each MSA. Finally, trees were visualized with the Python framework ETE3 [72].

### Gene expression and ion content assay

Tomato seeds were grown in sterile river sand, inoculated with either SynCom C, SA087, SA187, SA403, SA436 or SA444 and exposed to a 200 mM salt stress as described in Fig. S4. Plant shoot and root tissue were harvested at 4-, 7- and 10-days post salt imposition. The fresh shoot weight was recorded, and the root tissue was carefully extracted from the sand and vortexed briefly in phosphate buffer. All samples were frozen in liquid nitrogen and stored at −80 °C.

Five candidate genes for salt tolerance in tomato were chosen based on literature. The genes *HKT1;1*, *WRKY8*, *SOS1* and *SOS2* were previously shown to be associated with salt tolerance in tomato [40, 73–76]. The gene *CESA2* was also included as it showed a high homology to *AtCESA6*, which is important to salt tolerance in *Arabidopsis thaliana* [77]. Of these five genes, the primer sequences of *WRKY8* were taken from literature. For the others, custom primers were designed in silico on the RefSeq tomato assembly SL3.0 (GCF_000188115.4) with Geneious 11.1.5 (https://www.geneious.com). *Actin* was chosen as the housekeeping gene for normalization within samples [78]. Primer sequences, GC content, annealing temperature (Tm), expected amplicon length, NCBI GeneID, and references are shown in Table S3. Primer efficiencies were confirmed to be around 100% for all primer pairs using a range of serial dilutions.

Total RNA was extracted from the shoot and root tissue with the E.Z.N.A.^®^ Plant RNA kit (OMEGA BioTek). An optimal on-membrane DNase digestion step was performed during the RNA extraction by adding 10 μL DNase 1 and 70 μL Buffer RDD from the Qiagen DNase 1 kit. RNA quantity and purity as well as integrity were checked by Nanodrop and agarose gel electrophoresis. cDNA was synthesized from 300 and 600 ng of root and shoot RNA, respectively, with the iScript™ Select cDNA Synthesis kit (Bio-Rad). The synthesis reaction was carried out at 25 °C for 5 min, 46 °C for 20 min followed by 1 min at 95 °C. cDNA samples were diluted 5 times, aliquoted and stored at −20 °C. For the qPCR, each reaction contained: 5 μL IQ™ SYBR^®^, 2 μL cDNA and 1.5 μL of the forward and reverse primers with a total volume of 10 μL. Samples were loaded into a CFX Connect™ system (Bio-Rad) and initialized at 95 °C for 3 min, followed by 40 rounds of thermocycling. Each cycle started at 95 °C for 15 s, followed by 30 s at the primer-specific Tm (average temperature of the forward and reverse primers). Melting curves of all samples (from 55 °C to 95 °C in 5 second intervals with a 0.5 °C increment after each cycle) showed primer specificity and no primer-dimer formation. Relative expression of target genes was calculated for shoot and root tissue per timepoint as described previously [79]. Calculations were based on the control treated plants with salt as the reference group and Actin as the reference gene. Relative expression level (fold change) was calculated by the 2^-ΔΔCt method. Statistical significance was determined by a Dunnett’s test on the relative expression levels.

Ion concentrations was measured in tomato seedlings at ten days post salt imposition using an Ion Chromatography (IC) system 850 Professional (Metrohm, Switzerland), essentially as described previously [80]. Leaves of tomato seedlings were weighed in glass screw cap test tubes, dried and ashed in a furnace at 575 °C for 5 h. After cooling down to room temperature 1 mL formic acid (3 M) was added to each tube. Samples were heated at 103 °C for 15 min with shaking at 600 rpm. The extracts were then diluted by adding 9 mL of milliQ water to each sample. The samples were again heated and mixed at 80 °C for 30 min. After cooling down to room temperature all samples were measured in two dilutions 1/100 and 3/100 using a Metrohm 881 Compact IC pro ion chromatograph. Data was expressed in mg ion/mg dry weight.

A similar experiment was carried out with the same inoculums but in the non-sterile substrate. The shoot tissue of plants at ten days post salt imposition was harvested in order to record the fresh weight and to measure the ion content as described above.

## Supplementary information

Supplementary files

Table S1

Table S2

Table S3

Table S4

Table S5

Dataset S1

Dataset S2

Dataset S3

Dataset S4

## Data Availability

The raw sequencing reads are available upon request before they are uploaded to the Sequence Read Archive (SRA). The processed OTU/ASV tables and custom R and Python scripts are also available upon request.

## References

[1] Mendes R, Garbeva P, Raaijmakers JM (2013). The rhizosphere microbiome: significance of plant beneficial, plant pathogenic, and human pathogenic microorganisms. FEMS Microbiol Rev.

[2] Lemanceau P, Blouin M, Muller D, Moenne-Loccoz Y (2017). Let the core microbiota be functional. Trends Plant Sci.

[3] Mayak S, Tirosh T, Glick BR (2004). Plant growth-promoting bacteria confer resistance in tomato plants to salt stress. Plant Physiol Biochem.

[4] Yang J, Kloepper JW, Ryu CM (2009). Rhizosphere bacteria help plants tolerate abiotic stress. Trends Plant Sci.

[5] Maser P, Hosoo Y, Goshima S, Horie T, Eckelman B, Yamada K (2002). Glycine residues in potassium channel-like selectivity filters determine potassium selectivity in four-loop-per-subunit HKT transporters from plants. Proc Natl Acad Sci USA.

[6] Rus A, Lee BH, Munoz-Mayor A, Sharkhuu A, Miura K, Zhu JK (2004). AtHKT1 facilitates Na+ homeostasis and K+ nutrition in planta. Plant Physiol.

[7] Zhang H, Kim MS, Sun Y, Dowd SE, Shi H, Pare PW (2008). Soil bacteria confer plant salt tolerance by tissue-specific regulation of the sodium transporter HKT1. Mol Plant Microbe Interact.

[8] Saad MM, Eida AA, Hirt H (2020). Tailoring plant-associated microbial inoculants in agriculture: a roadmap for successful application. J Exp Bot.

[9] Schrire BD, Lavin M, Barker NP, Forest F (2009). Phylogeny of the tribe Indigofereae (Leguminosae–Papilionoideae): geographically structured more in succulent‐rich and temperate settings than in grass‐rich environments. Am J Bot.

[10] Lafi FF, Alam I, Geurts R, Bisseling T, Bajic VB, Hirt H (2016). Draft genome sequence of the phosphate-solubilizing bacterium pseudomonas argentinensis strain SA190 Isolated from the Desert Plant Indigofera argentea. Genome Announc.

[11] Andres-Barrao C, Lafi FF, Alam I, de Zelicourt A, Eida AA, Bokhari A (2017). Complete genome sequence analysis of enterobacter sp. SA187, a plant multi-stress tolerance promoting endophytic bacterium. Front Microbiol.

[12] Lafi FF, Alam I, Bisseling T, Geurts R, Bajic VB, Hirt H (2017). Draft genome sequence of the plant growth-promoting rhizobacterium acinetobacter radioresistens strain SA188 Isolated from the Desert Plant Indigofera argentea. Genome Announc.

[13] Lafi FF, Alam I, Geurts R, Bisseling T, Bajic VB, Hirt H (2017). Draft genome sequence of ochrobactrum intermedium strain SA148, a plant growth-promoting desert Rhizobacterium. Genome Announc.

[14] Baez-Rogelio A, Morales-Garcia YE, Quintero-Hernandez V, Munoz-Rojas J (2017). Next generation of microbial inoculants for agriculture and bioremediation. Micro Biotechnol.

[15] Bashan Y, de-Bashan LE, Prabhu S, Hernandez J-P (2014). Advances in plant growth-promoting bacterial inoculant technology: formulations and practical perspectives (1998–2013). Plant Soil.

[16] Raaijmakers JM. The minimal rhizosphere microbiome. Principles of plant-microbe interactions: Springer; 2015. 411–7.

[17] Lafi FF, Alam I, Geurts R, Bisseling T, Bajic VB, Hirt H (2017). Draft genome sequence of enterobacter sp. Sa187, an endophytic bacterium isolated from the desert plant indigofera argentea. Genome Announc.

[18] Zhou D, Huang X-F, Chaparro JM, Badri DV, Manter DK, Vivanco JM (2016). Root and bacterial secretions regulate the interaction between plants and PGPR leading to distinct plant growth promotion effects. Plant Soil.

[19] Park YG, Mun BG, Kang SM, Hussain A, Shahzad R, Seo CW (2017). Bacillus aryabhattai SRB02 tolerates oxidative and nitrosative stress and promotes the growth of soybean by modulating the production of phytohormones. PLoS ONE.

[20] Gran-Scheuch A, Trajkovic M, Parra L, Fraaije MW (2018). Mining the genome of streptomyces leeuwenhoekii: two new type I baeyer-villiger monooxygenases from atacama desert. Front Microbiol.

[21] Schneijderberg M, Cheng X, Franken C, de Hollander M, van Velzen R, Schmitz L (2020). Quantitative comparison between the rhizosphere effect of Arabidopsis thaliana and co-occurring plant species with a longer life history. ISME J.

[22] Rajput L, Imran A, Mubeen F, Hafeez FY (2013). Salt-tolerant PGPR strain Planococcus rifietoensis promotes the growth and yield of wheat (Triticum aestivum L.) cultivated in saline soil. Pak J Bot.

[23] Sharma S, Kulkarni J, Jha B (2016). Halotolerant rhizobacteria promote growth and enhance salinity tolerance in peanut. Front Microbiol.

[24] Singh RP, Jha PN (2016). The multifarious PGPR Serratia marcescens CDP-13 augments induced systemic resistance and enhanced salinity tolerance of wheat (Triticum aestivum L.). PLoS ONE.

[25] Sarkar A, Ghosh PK, Pramanik K, Mitra S, Soren T, Pandey S (2018). A halotolerant Enterobacter sp. displaying ACC deaminase activity promotes rice seedling growth under salt stress. Res Microbiol.

[26] Eida AA, Ziegler M, Lafi FF, Michell CT, Voolstra CR, Hirt H (2018). Desert plant bacteria reveal host influence and beneficial plant growth properties. PLoS ONE.

[27] Bokhari A, Essack M, Lafi FF, Andres-Barrao C, Jalal R, Alamoudi S (2019). Bioprospecting desert plant Bacillus endophytic strains for their potential to enhance plant stress tolerance. Sci Rep.

[28] Shekhawat K, Saad MM, Sheikh A, Mariappan K, Al-Mahmoudi H, Abdulhakim F (2021). Root endophyte induced plant thermotolerance by constitutive chromatin modification at heat stress memory gene loci. EMBO Rep.

[29] de Zélicourt A, Synek L, Saad MM, Alzubaidy H, Jalal R, Xie Y (2018). Ethylene induced plant stress tolerance by Enterobacter sp. SA187 is mediated by 2‐keto‐4‐methylthiobutyric acid production. PLoS Genet.

[30] Souza RD, Ambrosini A, Passaglia LM (2015). Plant growth-promoting bacteria as inoculants in agricultural soils. Genet Mol Biol.

[31] Vorholt JA, Vogel C, Carlstrom CI, Muller DB (2017). Establishing causality: opportunities of synthetic communities for plant microbiome research. Cell Host Microbe.

[32] Ahmad M, Zahir ZA, Asghar HN, Asghar M (2011). Inducing salt tolerance in mung bean through coinoculation with rhizobia and plant-growth-promoting rhizobacteria containing 1-aminocyclopropane-1-carboxylate deaminase. Can J Microbiol.

[33] Egamberdieva D, Wirth S, Jabborova D, Räsänen LA, Liao H (2017). Coordination between Bradyrhizobium and Pseudomonas alleviates salt stress in soybean through altering root system architecture. J Plant Interact.

[34] Finkel OM, Salas-Gonzalez I, Castrillo G, Conway JM, Law TF, Teixeira P (2020). A single bacterial genus maintains root growth in a complex microbiome. Nature.

[35] Flowers TJ, Munns R, Colmer TD (2015). Sodium chloride toxicity and the cellular basis of salt tolerance in halophytes. Ann Bot.

[36] Byrt CS, Munns R, Burton RA, Gilliham M, Wege S (2018). Root cell wall solutions for crop plants in saline soils. Plant Sci.

[37] Ma L, Zhang H, Sun L, Jiao Y, Zhang G, Miao C (2012). NADPH oxidase AtrbohD and AtrbohF function in ROS-dependent regulation of Na+/K+ homeostasis in Arabidopsis under salt stress. J Exp Bot.

[38] Wu H, Shabala L, Zhou M, Su N, Wu Q, Ul‐Haq T (2019). Root vacuolar Na+ sequestration but not exclusion from uptake correlates with barley salt tolerance. Plant J.

[39] Ji H, Pardo JM, Batelli G, Van Oosten MJ, Bressan RA, Li X (2013). The Salt Overly Sensitive (SOS) pathway: established and emerging roles. Mol Plant.

[40] Olias R, Eljakaoui Z, Pardo JM, Belver A (2009). The Na(+)/H(+) exchanger SOS1 controls extrusion and distribution of Na(+) in tomato plants under salinity conditions. Plant Signal Behav.

[41] Olias R, Eljakaoui Z, Li J, De Morales PA, Marin‐Manzano MC, Pardo JM (2009). The plasma membrane Na+/H+ antiporter SOS1 is essential for salt tolerance in tomato and affects the partitioning of Na+ between plant organs. Plant Cell Environ.

[42] Horie T, Yoshida K, Nakayama H, Yamada K, Oiki S, Shinmyo A (2001). Two types of HKT transporters with different properties of Na+ and K+ transport in Oryza sativa. Plant J.

[43] Moller IS, Gilliham M, Jha D, Mayo GM, Roy SJ, Coates JC (2009). Shoot Na+ exclusion and increased salinity tolerance engineered by cell type-specific alteration of Na+ transport in Arabidopsis. Plant Cell.

[44] Plett D, Safwat G, Gilliham M, Skrumsager Møller I, Roy S, Shirley N (2010). Improved salinity tolerance of rice through cell type-specific expression of AtHKT1; 1. PloS ONE.

[45] Zhao C, Zhang H, Song C, Zhu JK, Shabala S (2020). Mechanisms of plant responses and adaptation to soil salinity. Innov (N. Y).

[46] Muller DB, Vogel C, Bai Y, Vorholt JA (2016). The plant microbiota: systems-level insights and perspectives. Annu Rev Genet.

[47] Naylor D, DeGraaf S, Purdom E, Coleman-Derr D (2017). Drought and host selection influence bacterial community dynamics in the grass root microbiome. ISME J.

[48] Faust K, Raes J (2012). Microbial interactions: from networks to models. Nat Rev Microbiol.

[49] Silliker J. H. Microb Ecol Foods, Vol I Factors Affect Life Death Microorg. 1980;54–5.

[50] Awlia M, Nigro A, Fajkus J, Schmoeckel SM, Negrão S, Santelia D (2016). High-throughput non-destructive phenotyping of traits that contribute to salinity tolerance in Arabidopsis thaliana. Front Plant Sci.

[51] Lundberg DS, Lebeis SL, Paredes SH, Yourstone S, Gehring J, Malfatti S (2012). Defining the core Arabidopsis thaliana root microbiome. Nature.

[52] Edgar RC (2013). UPARSE: highly accurate OTU sequences from microbial amplicon reads. Nat Methods.

[53] Rognes T, Flouri T, Nichols B, Quince C, Mahe F (2016). VSEARCH: a versatile open source tool for metagenomics. PeerJ.

[54] Quast C, Pruesse E, Yilmaz P, Gerken J, Schweer T, Yarza P (2013). The SILVA ribosomal RNA gene database project: improved data processing and web-based tools. Nucleic Acids Res.

[55] Callahan B, McMurdie P, Rosen M, Han A, Johnson AJA, Holmes SP (2016). 2016. DADA2: high-resolution sample inference from Illumina amplicon data. Nat Methods.

[56] Wang Q, Garrity GM, Tiedje JM, Cole JR (2007). Naive Bayesian classifier for rapid assignment of rRNA sequences into the new bacterial taxonomy. Appl Environ Microbiol.

[57] Paulson JN, Stine OC, Bravo HC, Pop M (2013). Differential abundance analysis for microbial marker-gene surveys. Nat Methods.

[58] Hartman K, van der Heijden MGA, Wittwer RA, Banerjee S, Walser J, Schlaeppi K (2018). Cropping practices manipulate abundance patterns of root and soil microbiome members paving the way to smart farming. Microbiome.

[59] Hongoh Y, Yuzawa H, Ohkuma M, Kudo T (2003). Evaluation of primers and PCR conditions for the analysis of 16S rRNA genes from a natural environment. FEMS Microbiol Lett.

[60] Selvakumar G, Krishnamoorthy R, Kim K, Sa TM (2016). Genetic diversity and association characters of bacteria isolated from arbuscular mycorrhizal fungal spore walls. PLoS ONE.

[61] Bolger AM, Lohse M, Usadel B (2014). Trimmomatic: a flexible trimmer for Illumina sequence data. Bioinformatics.

[62] Bankevich A, Nurk S, Antipov D, Gurevich AA, Dvorkin M, Kulikov AS (2012). SPAdes: a new genome assembly algorithm and its applications to single-cell sequencing. J Comput Biol.

[63] Parks DH, Imelfort M, Skennerton CT, Hugenholtz P, Tyson GW (2015). CheckM: assessing the quality of microbial genomes recovered from isolates, single cells, and metagenomes. Genome Res.

[64] Pedregosa F, Varoquaux G, Gramfort A, Michel V, Thirion B, Grisel O (2011). Scikit-learn: machine learning in Python. J Mach Learn Res.

[65] Gurevich A, Saveliev V, Vyahhi N, Tesler G (2013). QUAST: quality assessment tool for genome assemblies. Bioinformatics.

[66] Hyatt D, Chen GL, Locascio PF, Land ML, Larimer FW, Hauser LJ (2010). Prodigal: prokaryotic gene recognition and translation initiation site identification. BMC Bioinforma.

[67] Wu M, Scott AJ (2012). Phylogenomic analysis of bacterial and archaeal sequences with AMPHORA2. Bioinformatics.

[68] Eddy SR (2011). Accelerated profile HMM searches. PLoS Comput Biol.

[69] Madeira F, Park YM, Lee J, Buso N, Gur T, Madhusoodanan N (2019). The EMBL-EBI search and sequence analysis tools APIs in 2019. Nucleic Acids Res.

[70] Nguyen LT, Schmidt HA, von Haeseler A, Minh BQ (2015). IQ-TREE: a fast and effective stochastic algorithm for estimating maximum-likelihood phylogenies. Mol Biol Evol.

[71] Kalyaanamoorthy S, Minh BQ, Wong TKF, von Haeseler A, Jermiin LS (2017). ModelFinder: fast model selection for accurate phylogenetic estimates. Nat Methods.

[72] Huerta-Cepas J, Serra F, Bork P (2016). ETE 3: reconstruction, analysis, and visualization of phylogenomic data. Mol Biol Evol.

[73] Kou X, Chen X, Mao C, He Y, Feng Y, Wu C (2019). Selection and mechanism exploration for salt-tolerant genes in tomato. J Horticultural Sci Biotechnol.

[74] Rus A, Yokoi S, Sharkhuu A, Reddy M, Lee B-h, Matsumoto TK (2001). AtHKT1 is a salt tolerance determinant that controls Na+ entry into plant roots. Proc Natl Acad Sci.

[75] Gao YF, Liu JK, Yang FM, Zhang GY, Wang D, Zhang L (2020). The WRKY transcription factor WRKY8 promotes resistance to pathogen infection and mediates drought and salt stress tolerance in Solanum lycopersicum. Physiol Plant.

[76] Huertas R, Olias R, Eljakaoui Z, Galvez FJ, Li J, De Morales PA (2012). Overexpression of SlSOS2 (SlCIPK24) confers salt tolerance to transgenic tomato. Plant Cell Environ.

[77] Zhang SS, Sun L, Dong X, Lu SJ, Tian W, Liu JX (2016). Cellulose synthesis genes CESA6 and CSI1 are important for salt stress tolerance in Arabidopsis. J Integr Plant Biol.

[78] Lovdal T, Lillo C (2009). Reference gene selection for quantitative real-time PCR normalization in tomato subjected to nitrogen, cold, and light stress. Anal Biochem.

[79] Livak KJ, Schmittgen TD (2001). Analysis of relative gene expression data using real-time quantitative PCR and the 2(-Delta Delta C(T)) Method. Methods.

[80] Roman VJ, den Toom LA, Gamiz CC, van der Pijl N, Visser RG, van Loo EN (2020). Differential responses to salt stress in ion dynamics, growth and seed yield of European quinoa varieties. Environ Exp Bot.

