# Supplementary material for: Synthetic bacterial community derived from a desert rhizosphere confers salt stress resilience to tomato in the presence of a soil microbiome
Source: ISME J. 2022 Apr 20;16(8):1907–20. doi: 10.1038/s41396-022-01238-3 (PMC9296610; doi:10.1038/s41396-022-01238-3)

Supporting information for

A synthetic bacterial community derived from a desert rhizosphere confers salt stress resilience to tomato in the presence of a soil microbiome

Lucas Schmitz#, Zhichun Yan#, Martinus Schneijderberg, Martijn de Roij, Rick Pijnenburg, Qi Zheng, Carolien Franken, Annemarie Dechesne, Luisa M. Trindade, Robin van Velzen, Ton Bisseling, Rene Geurts*, and Xu Cheng*
corresponding authors: rene.geurts@wur.nl, xu.cheng@wur.nl

Figures

Fig. S1: Former agricultural areas in the Jizan desert, which suffer from high salinity, are scarcely populated with indigofera.

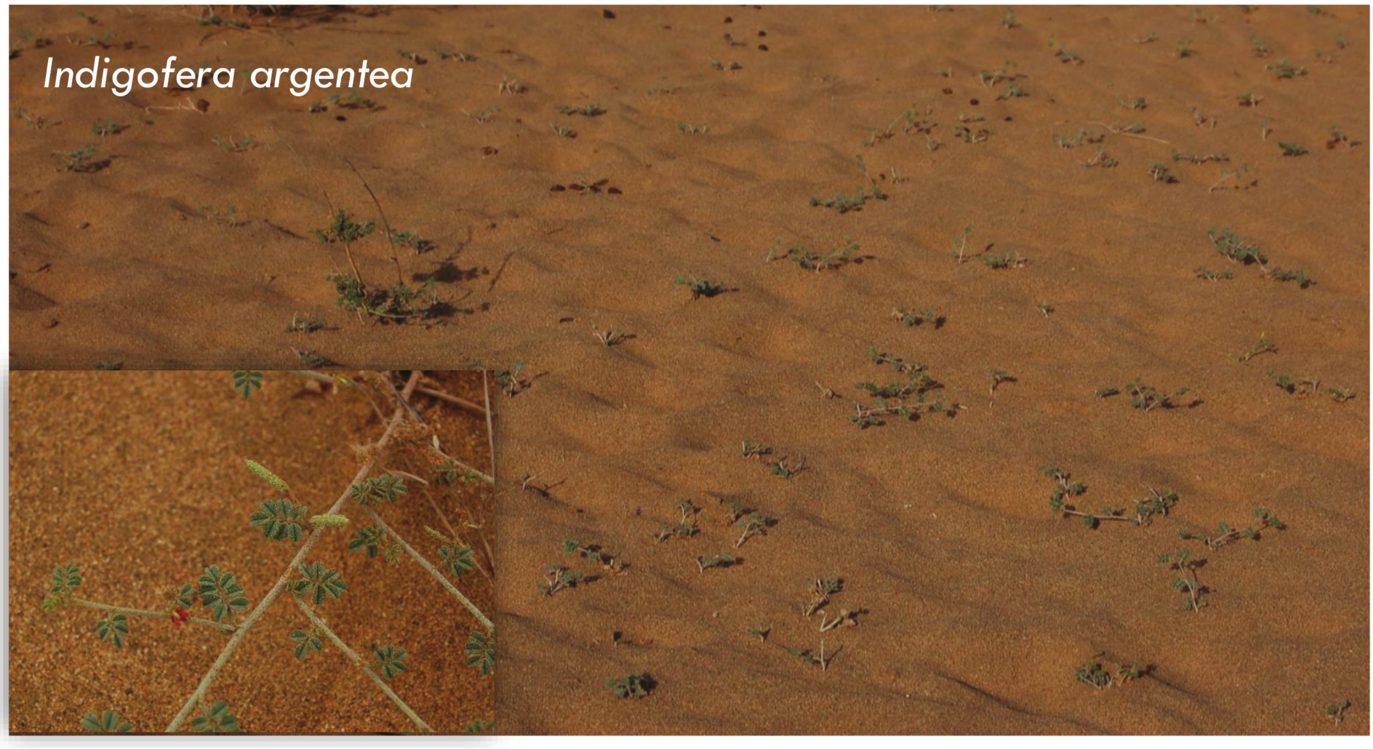

Fig. S2: Venn diagram showing number of depleted OTUs in the Rhizo and EC of *I. argentea* in comparison to the Soil. See Dataset S2.

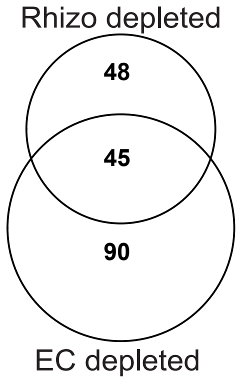

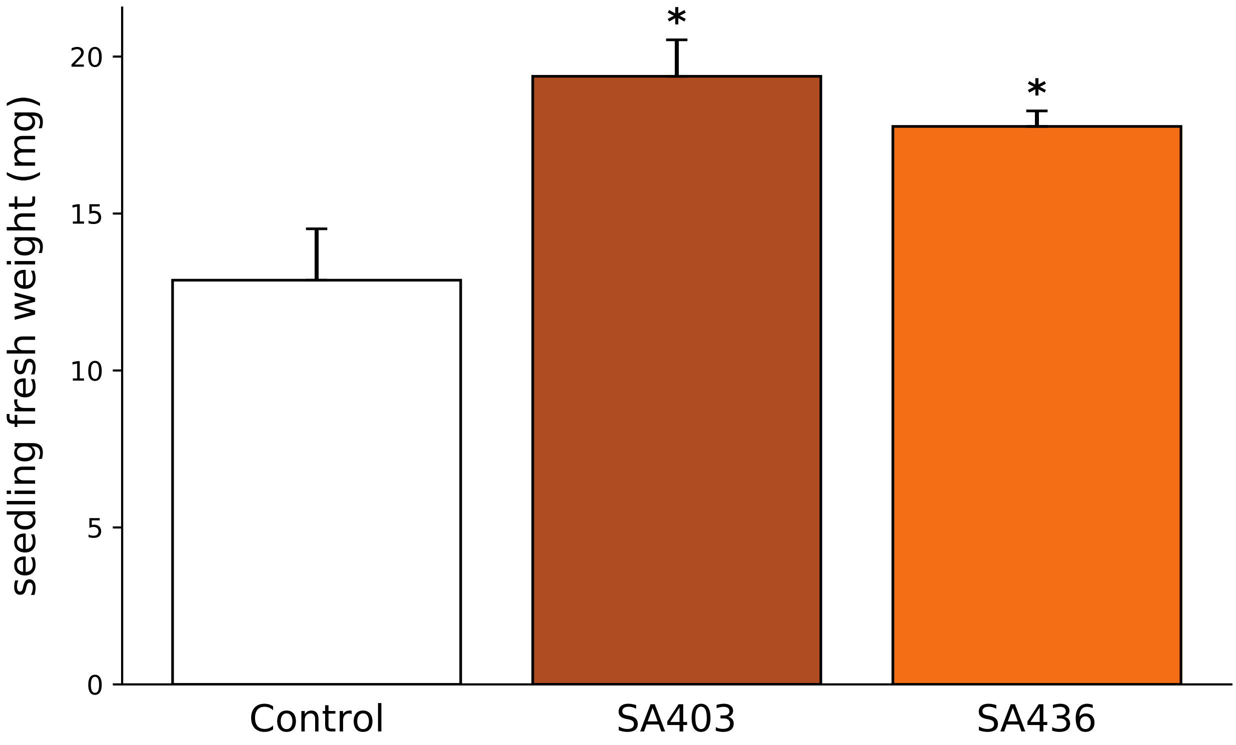

Fig. S3 : Growth promotion of arabidopsis by the Jizan strains *Ensifer* sp. SA403 and *Bacillus* sp. SA436. Ten arabidopsis seeds were grown on a plate which was the experimental unit. There were four experimental units per inoculation which also included a sterile control. Seedling fresh weight was defined as the average weight per experimental unit and determined eleven days post bacterial inoculation. The y-axis shows the average seedling fresh weights with the standard deviation as error bars. An asterisk is a significant difference ($p < 0.05$) from the control per Dunnett's test. Strains SA436 and SA403 were grown in 5 mL 1/10th TSB or 5 mL YEM liquid medium, respectively, for 48 hours at 28°C. Bacterial cells were washed and resuspended in 10 mM $MgSO_4$ and the final suspension had an OD_{600} of 1.0 (~109 cfu/mL). Seeds of *Arabidopsis thaliana* Columbia-0 were surface steriled by washing with ethanol and soaking in 1/4 strength commercial bleach for 10 minutes. Seeds were transferred to wet filter paper in Petri dishes. After incubating at 4°C for 3 days, 10 seeds were sown on plates containing 50 mL 1/2th Murashige Skoog medium. Two µL of the bacterial suspension was applied to the root tips of one-week-old seedlings. Control plants were inoculated with 2 µL of 10 mM $MgSO_4$.

Fig. S4: Maximum likelihood trees based on the AMPHORA gene alignments of nine different genera. (a) *Massilia*, (b) *Streptomyces*, (c) *Ensifer*, (d) *Bacillus*, (e) *Enterobacter*, (f) *Acinetobacter*, (g) *Ochrobactrum*, (h) *Pseudomonas*, (i) *Ralstonia*. Each tree contains at least one of the strains found in the Jizan SynCom, representative genomes and plant-associated strains for that genus. All trees are rooted on a suitable outgroup and the Jizan strains are colored in red. The distance scale indicates the number of differences between sequences.

a

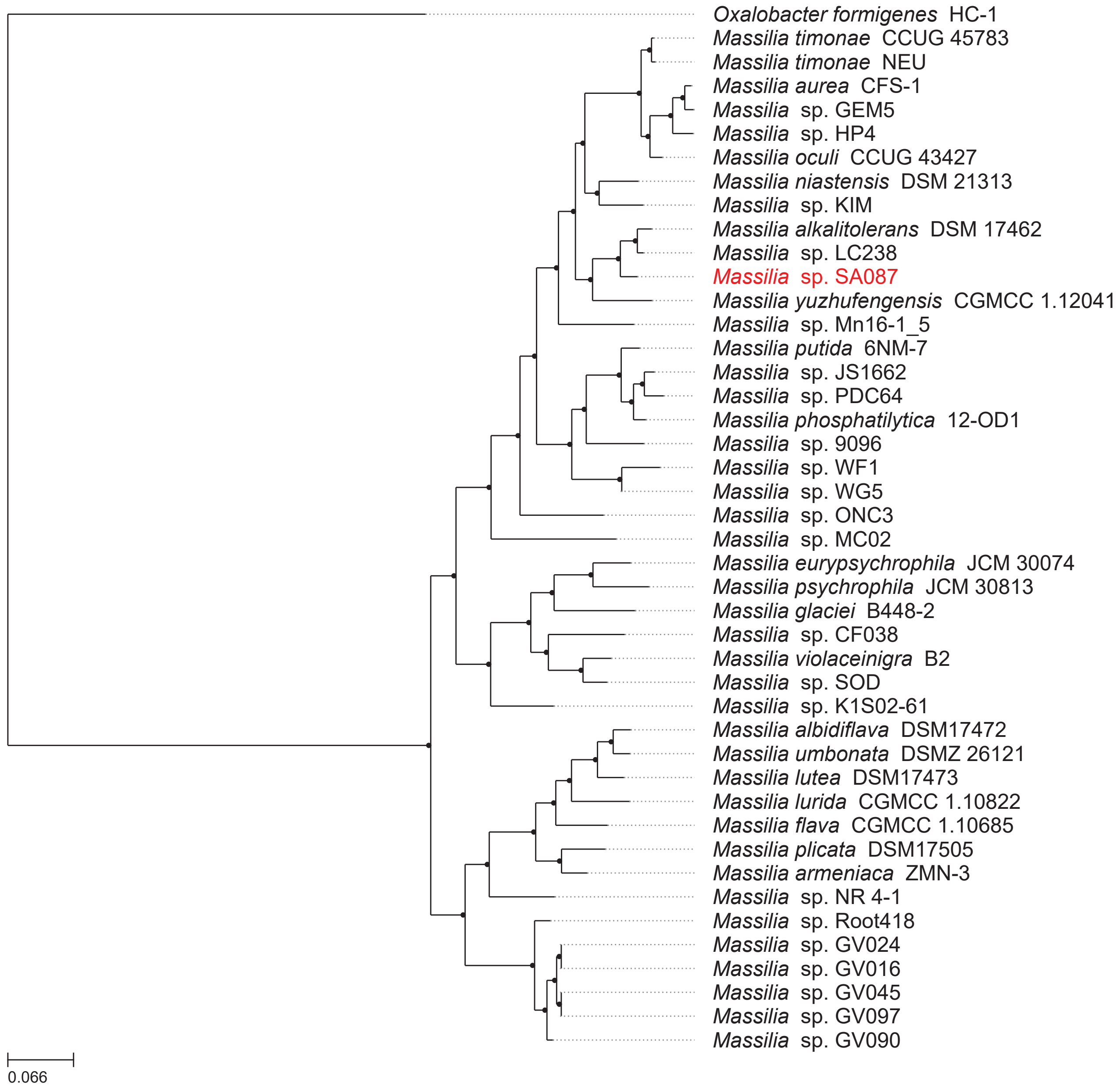

b

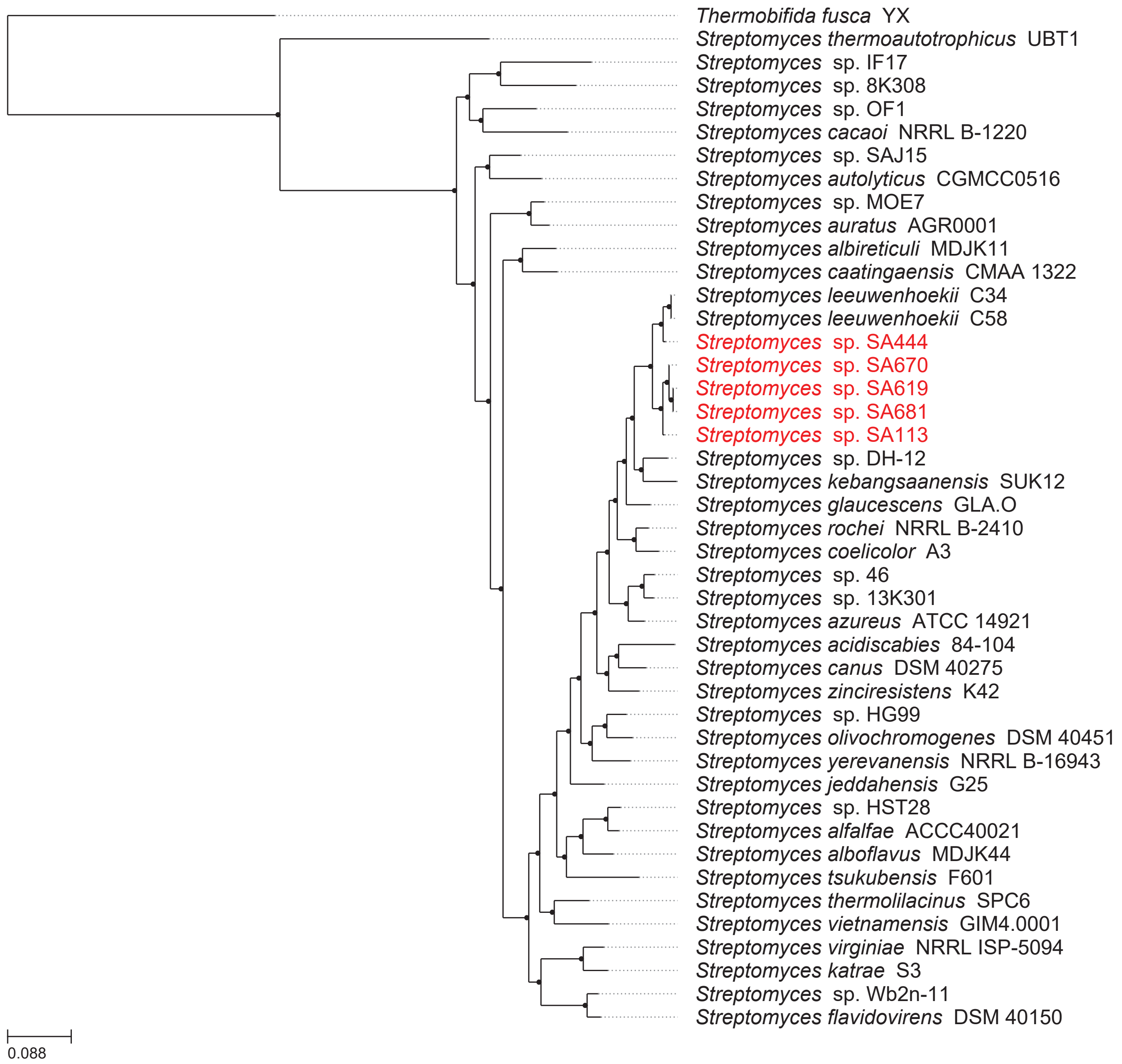
Thermobifida fusca YX
Streptomyces thermoautotrophicus UBT1
Streptomyces sp. IF17
Streptomyces sp. 8K308
Streptomyces sp. OF1
Streptomyces cacaoi NRRL B-1220
Streptomyces sp. SAJ15
Streptomyces autolyticus CGMCC0516
Streptomyces sp. MOE7
Streptomyces auratus AGR0001
Streptomyces albireticuli MDJK11
Streptomyces caatingaensis CMAA 1322
Streptomyces leeuwenhoekii C34
Streptomyces leeuwenhoekii C58
Streptomyces sp. SA444
Streptomyces sp. SA670
Streptomyces sp. SA619
Streptomyces sp. SA681
Streptomyces sp. SA113
Streptomyces sp. DH-12
Streptomyces kebangsaanensis SUK12
Streptomyces glaucescens GLA.O
Streptomyces rochei NRRL B-2410
Streptomyces coelicolor A3
Streptomyces sp. 46
Streptomyces sp. 13K301
Streptomyces azureus ATCC 14921
Streptomyces acidiscabies 84-104
Streptomyces canus DSM 40275
Streptomyces zinciresistens K42
Streptomyces sp. HG99
Streptomyces olivochromogenes DSM 40451
Streptomyces yerevanensis NRRL B-16943
Streptomyces jeddahensis G25
Streptomyces sp. HST28
Streptomyces alfalfae ACCC40021
Streptomyces alboflavus MDJK44
Streptomyces tsukubensis F601
Streptomyces thermolilacinus SPC6
Streptomyces vietnamensis GIM4.0001
Streptomyces virginiae NRRL ISP-5094
Streptomyces katrae S3
Streptomyces sp. Wb2n-11
Streptomyces flavidovirens DSM 40150
0.088

c

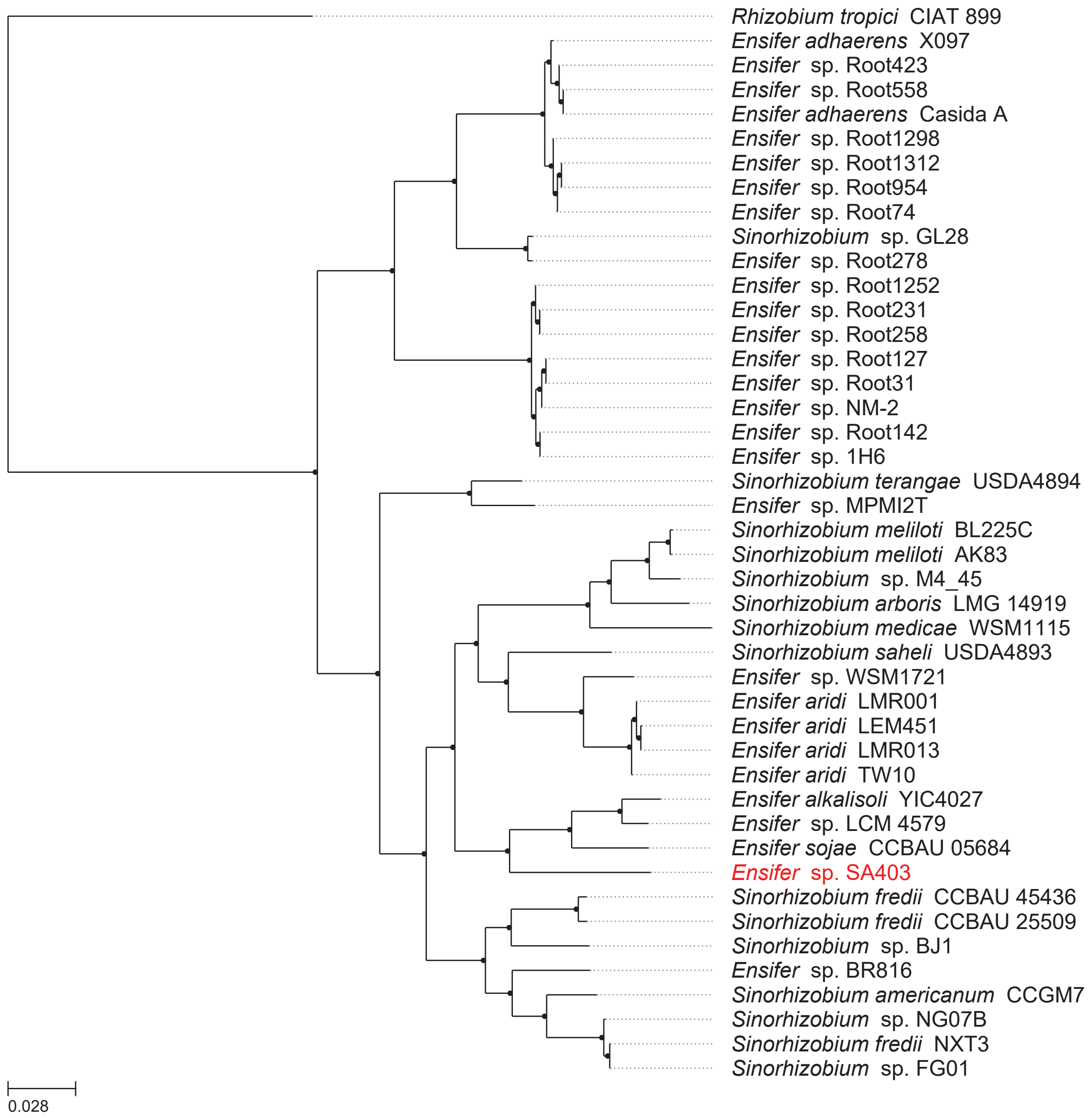
Rhizobium tropici CIAT 899
Ensifer adhaerens X097
Ensifer sp. Root423
Ensifer sp. Root558
Ensifer adhaerens Casida A
Ensifer sp. Root1298
Ensifer sp. Root1312
Ensifer sp. Root954
Ensifer sp. Root74
Sinorhizobium sp. GL28
Ensifer sp. Root278
Ensifer sp. Root1252
Ensifer sp. Root231
Ensifer sp. Root258
Ensifer sp. Root127
Ensifer sp. Root31
Ensifer sp. NM-2
Ensifer sp. Root142
Ensifer sp. 1H6
Sinorhizobium terangae USDA4894
Ensifer sp. MPMI2T
Sinorhizobium meliloti BL225C
Sinorhizobium meliloti AK83
Sinorhizobium sp. M4_45
Sinorhizobium arboris LMG 14919
Sinorhizobium medicae WSM1115
Sinorhizobium saheli USDA4893
Ensifer sp. WSM1721
Ensifer aridi LMR001
Ensifer aridi LEM451
Ensifer aridi LMR013
Ensifer aridi TW10
Ensifer alkalisoli YIC4027
Ensifer sp. LCM 4579
Ensifer sojae CCBAU 05684
Ensifer sp. SA403
Sinorhizobium fredii CCBAU 45436
Sinorhizobium fredii CCBAU 25509
Sinorhizobium sp. BJ1
Ensifer sp. BR816
Sinorhizobium americanum CCGM7
Sinorhizobium sp. NG07B
Sinorhizobium fredii NXT3
Sinorhizobium sp. FG01
0.028

d

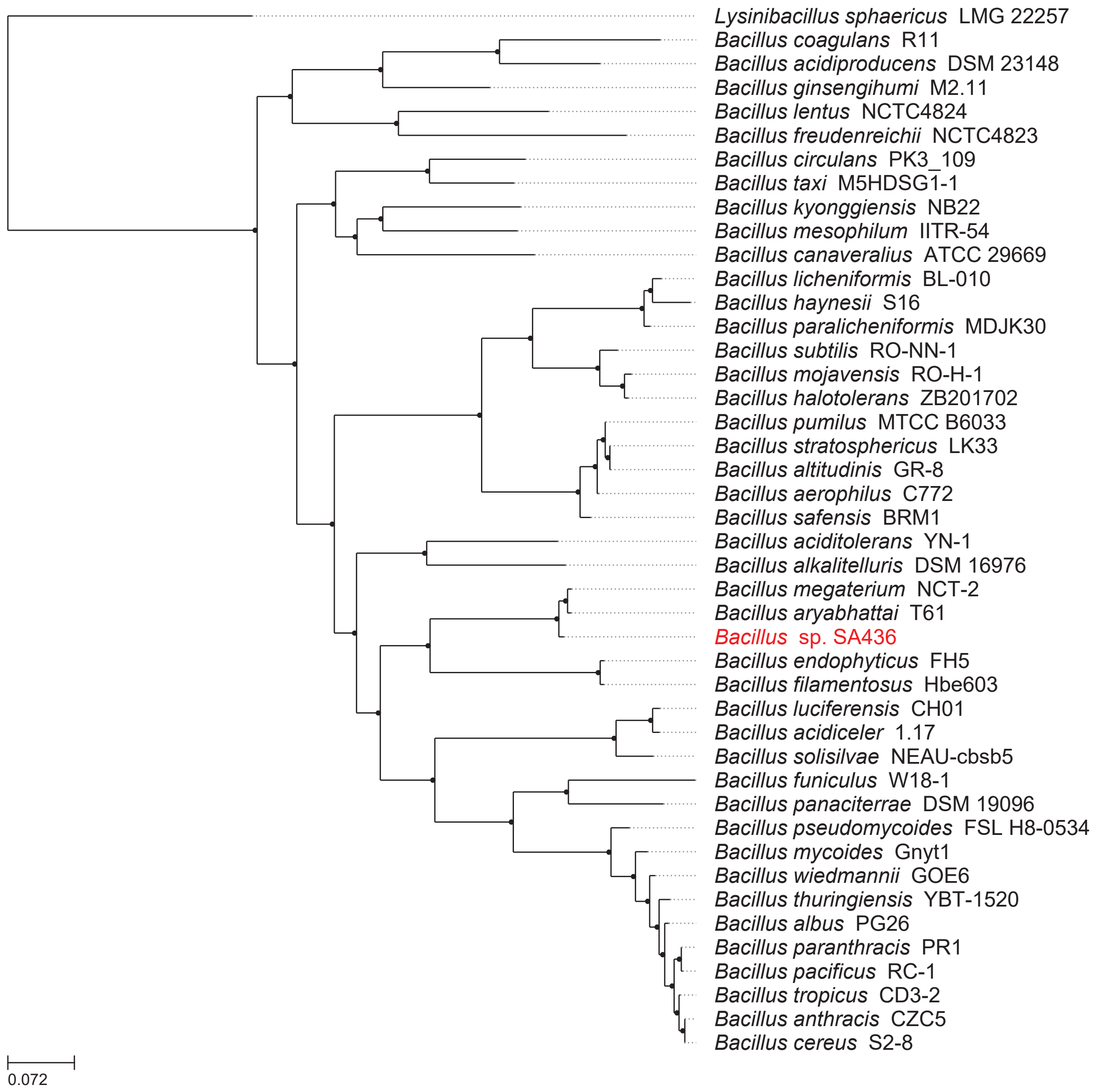
Lysinibacillus sphaericus LMG 22257
Bacillus coagulans R11
Bacillus acidiproducens DSM 23148
Bacillus ginsengihumi M2.11
Bacillus lentus NCTC4824
Bacillus freudenreichii NCTC4823
Bacillus circulans PK3_109
Bacillus taxi M5HDSG1-1
Bacillus kyonggiensis NB22
Bacillus mesophilum IITR-54
Bacillus canaveralius ATCC 29669
Bacillus licheniformis BL-010
Bacillus haynesii S16
Bacillus paralicheniformis MDJK30
Bacillus subtilis RO-NN-1
Bacillus mojavensis RO-H-1
Bacillus halotolerans ZB201702
Bacillus pumilus MTCC B6033
Bacillus stratosphericus LK33
Bacillus altitudinis GR-8
Bacillus aerophilus C772
Bacillus safensis BRM1
Bacillus aciditolerans YN-1
Bacillus alkalitelluris DSM 16976
Bacillus megaterium NCT-2
Bacillus aryabhattai T61
Bacillus sp. SA436
Bacillus endophyticus FH5
Bacillus filamentosus Hbe603
Bacillus luciferensis CH01
Bacillus acidiceler 1.17
Bacillus solisilvae NEAU-cbsb5
Bacillus funiculus W18-1
Bacillus panaciterrae DSM 19096
Bacillus pseudomycoides FSL H8-0534
Bacillus mycoides Gnyt1
Bacillus wiedmannii GOE6
Bacillus thuringiensis YBT-1520
Bacillus albus PG26
Bacillus paranthracis PR1
Bacillus pacificus RC-1
Bacillus tropicus CD3-2
Bacillus anthracis CZC5
Bacillus cereus S2-8
0.072

e

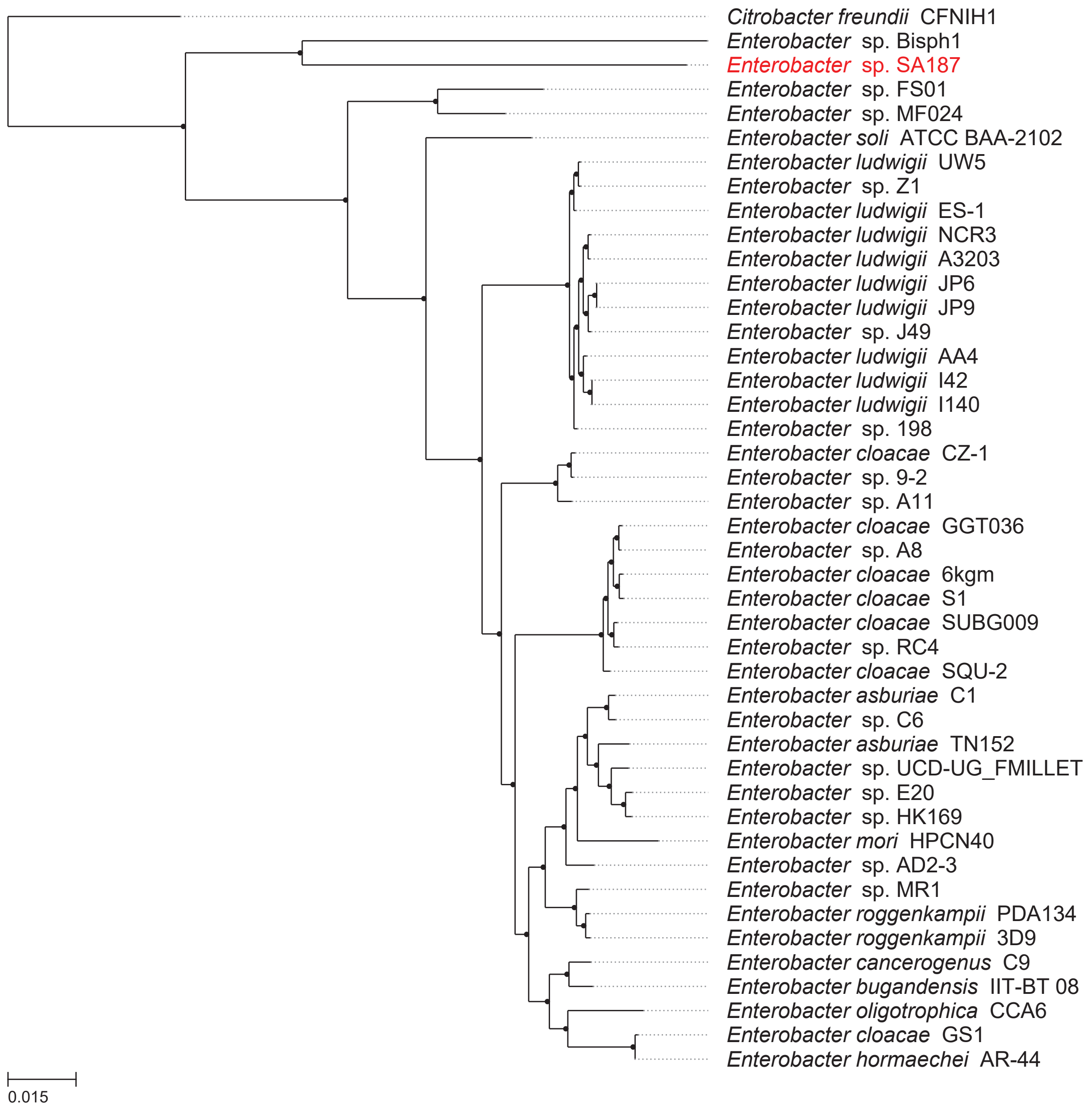

Citrobacter freundii CFNIH1
Enterobacter sp. Bisph1
Enterobacter sp. SA187
Enterobacter sp. FS01
Enterobacter sp. MF024
Enterobacter soli ATCC BAA-2102
Enterobacter ludwigii UW5
Enterobacter sp. Z1
Enterobacter ludwigii ES-1
Enterobacter ludwigii NCR3
Enterobacter ludwigii A3203
Enterobacter ludwigii JP6
Enterobacter ludwigii JP9
Enterobacter sp. J49
Enterobacter ludwigii AA4
Enterobacter ludwigii I42
Enterobacter ludwigii I140
Enterobacter sp. 198
Enterobacter cloacae CZ-1
Enterobacter sp. 9-2
Enterobacter sp. A11
Enterobacter cloacae GGT036
Enterobacter sp. A8
Enterobacter cloacae 6kgm
Enterobacter cloacae S1
Enterobacter cloacae SUBG009
Enterobacter sp. RC4
Enterobacter cloacae SQU-2
Enterobacter asburiae C1
Enterobacter sp. C6
Enterobacter asburiae TN152
Enterobacter sp. UCD-UG_FMILLET
Enterobacter sp. E20
Enterobacter sp. HK169
Enterobacter mori HPCN40
Enterobacter sp. AD2-3
Enterobacter sp. MR1
Enterobacter roggenkampii PDA134
Enterobacter roggenkampii 3D9
Enterobacter cancerogenus C9
Enterobacter bugandensis IIT-BT 08
Enterobacter oligotrophica CCA6
Enterobacter cloacae GS1
Enterobacter hormaechei AR-44
0.015

f

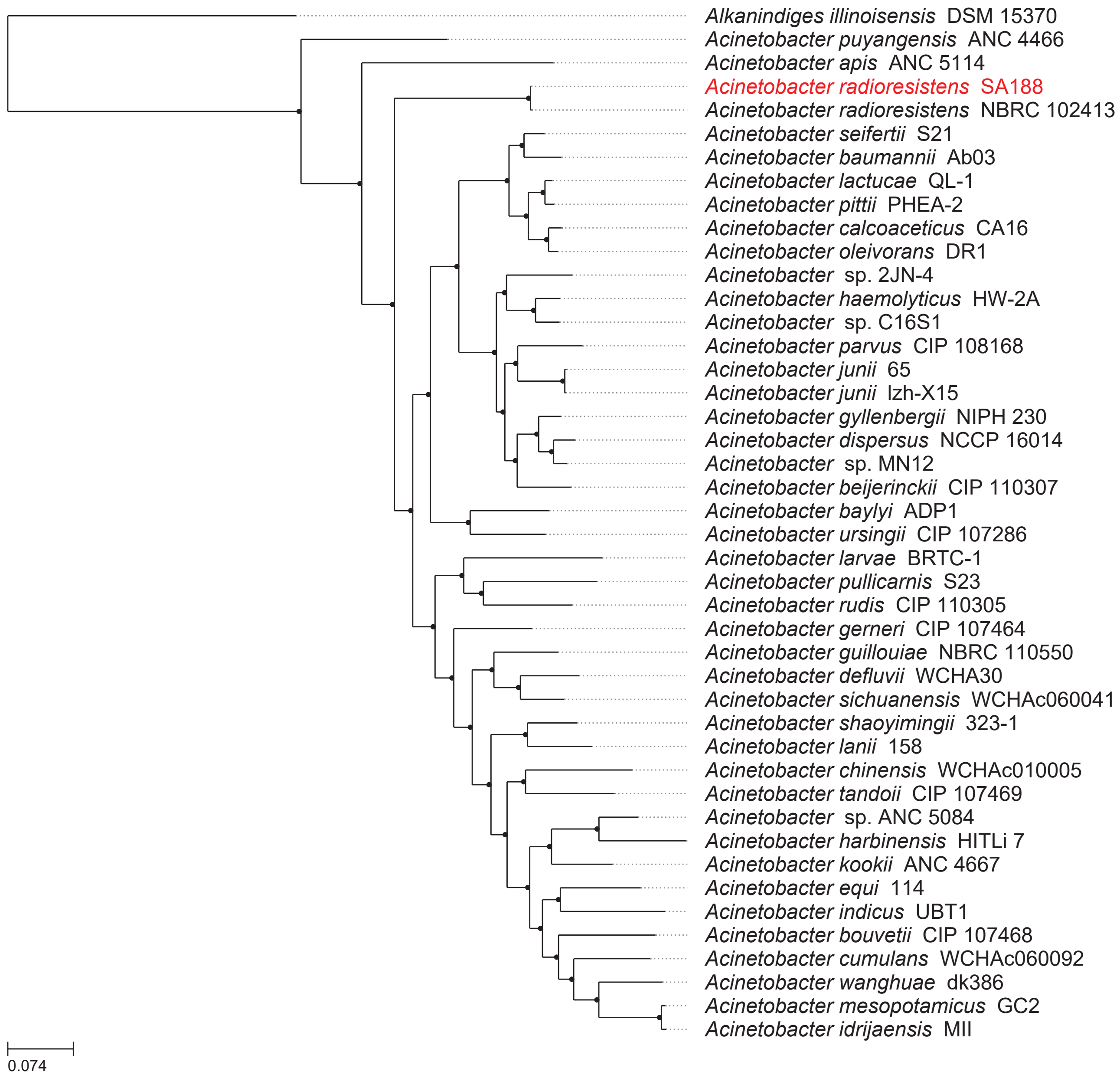

Alkanindiges illinoisensis DSM 15370
Acinetobacter puyangensis ANC 4466
Acinetobacter apis ANC 5114
Acinetobacter radioresistens SA188
Acinetobacter radioresistens NBRC 102413
Acinetobacter seifertii S21
Acinetobacter baumannii Ab03
Acinetobacter lactucae QL-1
Acinetobacter pittii PHEA-2
Acinetobacter calcoaceticus CA16
Acinetobacter oleivorans DR1
Acinetobacter sp. 2JN-4
Acinetobacter haemolyticus HW-2A
Acinetobacter sp. C16S1
Acinetobacter parvus CIP 108168
Acinetobacter junii 65
Acinetobacter junii lzh-X15
Acinetobacter gyllenbergii NIPH 230
Acinetobacter dispersus NCCP 16014
Acinetobacter sp. MN12
Acinetobacter beijerinckii CIP 110307
Acinetobacter baylyi ADP1
Acinetobacter ursingii CIP 107286
Acinetobacter larvae BRTC-1
Acinetobacter pullicarnis S23
Acinetobacter rudis CIP 110305
Acinetobacter gerneri CIP 107464
Acinetobacter guillouiae NBRC 110550
Acinetobacter defluvii WCHA30
Acinetobacter sichuanensis WCHAc060041
Acinetobacter shaoyimingii 323-1
Acinetobacter lanii 158
Acinetobacter chinensis WCHAc010005
Acinetobacter tandoii CIP 107469
Acinetobacter sp. ANC 5084
Acinetobacter harbinensis HITLi 7
Acinetobacter kookii ANC 4667
Acinetobacter equi 114
Acinetobacter indicus UBT1
Acinetobacter bouvetii CIP 107468
Acinetobacter cumulans WCHAc060092
Acinetobacter wanghuae dk386
Acinetobacter mesopotamicus GC2
Acinetobacter idrijaensis MII
0.074

g

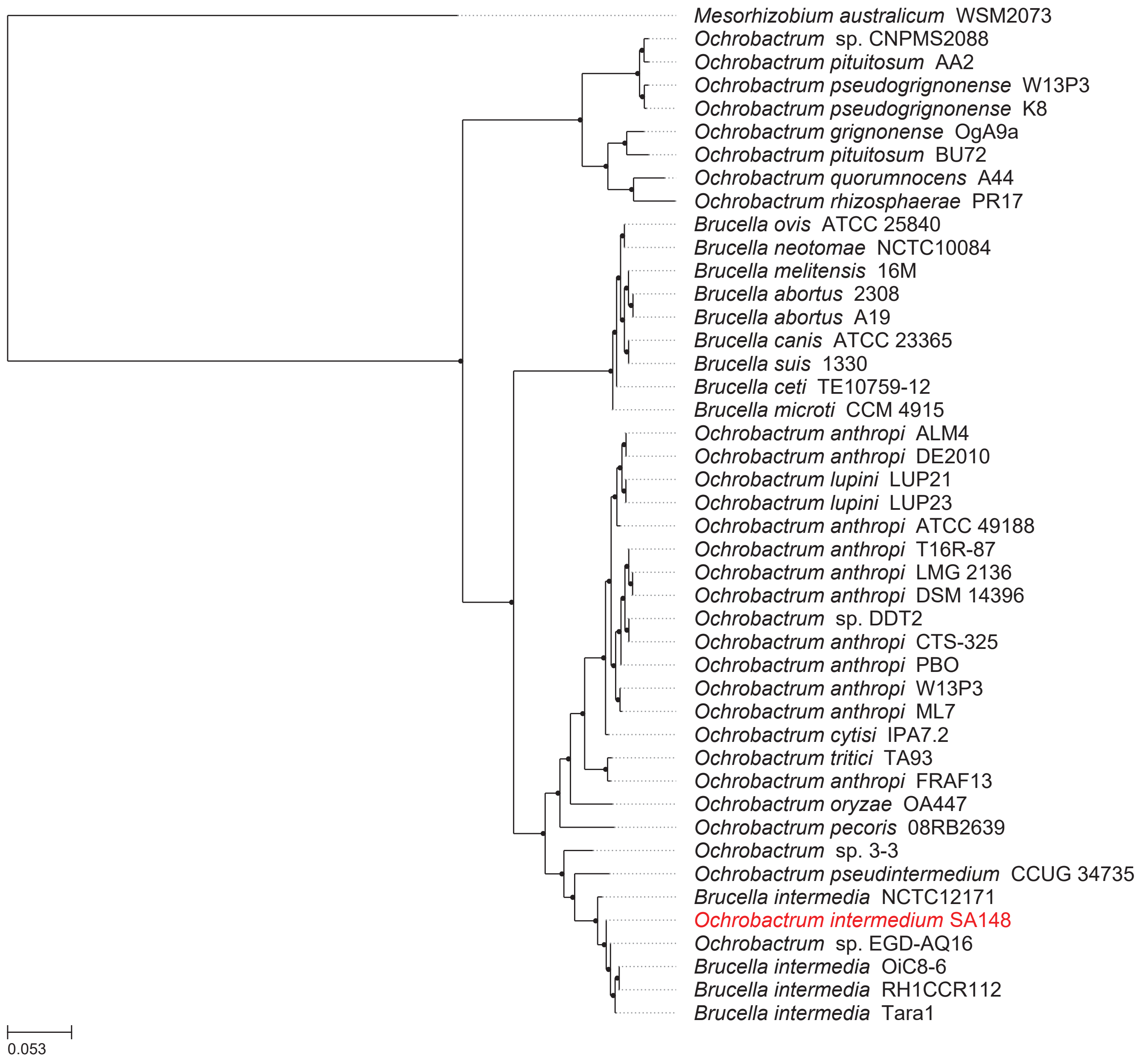

Mesorhizobium australicum WSM2073
Ochrobactrum sp. CNPMS2088
Ochrobactrum pituitosum AA2
Ochrobactrum pseudogrignonense W13P3
Ochrobactrum pseudogrignonense K8
Ochrobactrum grignonense OgA9a
Ochrobactrum pituitosum BU72
Ochrobactrum quorumnocens A44
Ochrobactrum rhizosphaerae PR17
Brucella ovis ATCC 25840
Brucella neotomae NCTC10084
Brucella melitensis 16M
Brucella abortus 2308
Brucella abortus A19
Brucella canis ATCC 23365
Brucella suis 1330
Brucella ceti TE10759-12
Brucella microti CCM 4915
Ochrobactrum anthropi ALM4
Ochrobactrum anthropi DE2010
Ochrobactrum lupini LUP21
Ochrobactrum lupini LUP23
Ochrobactrum anthropi ATCC 49188
Ochrobactrum anthropi T16R-87
Ochrobactrum anthropi LMG 2136
Ochrobactrum anthropi DSM 14396
Ochrobactrum sp. DDT2
Ochrobactrum anthropi CTS-325
Ochrobactrum anthropi PBO
Ochrobactrum anthropi W13P3
Ochrobactrum anthropi ML7
Ochrobactrum cytisi IPA7.2
Ochrobactrum tritici TA93
Ochrobactrum anthropi FRAF13
Ochrobactrum oryzae OA447
Ochrobactrum pecoris 08RB2639
Ochrobactrum sp. 3-3
Ochrobactrum pseudintermedium CCUG 34735
Brucella intermedia NCTC12171
Ochrobactrum intermedium SA148
Ochrobactrum sp. EGD-AQ16
Brucella intermedia OiC8-6
Brucella intermedia RH1CCR112
Brucella intermedia Tara1
0.053

h

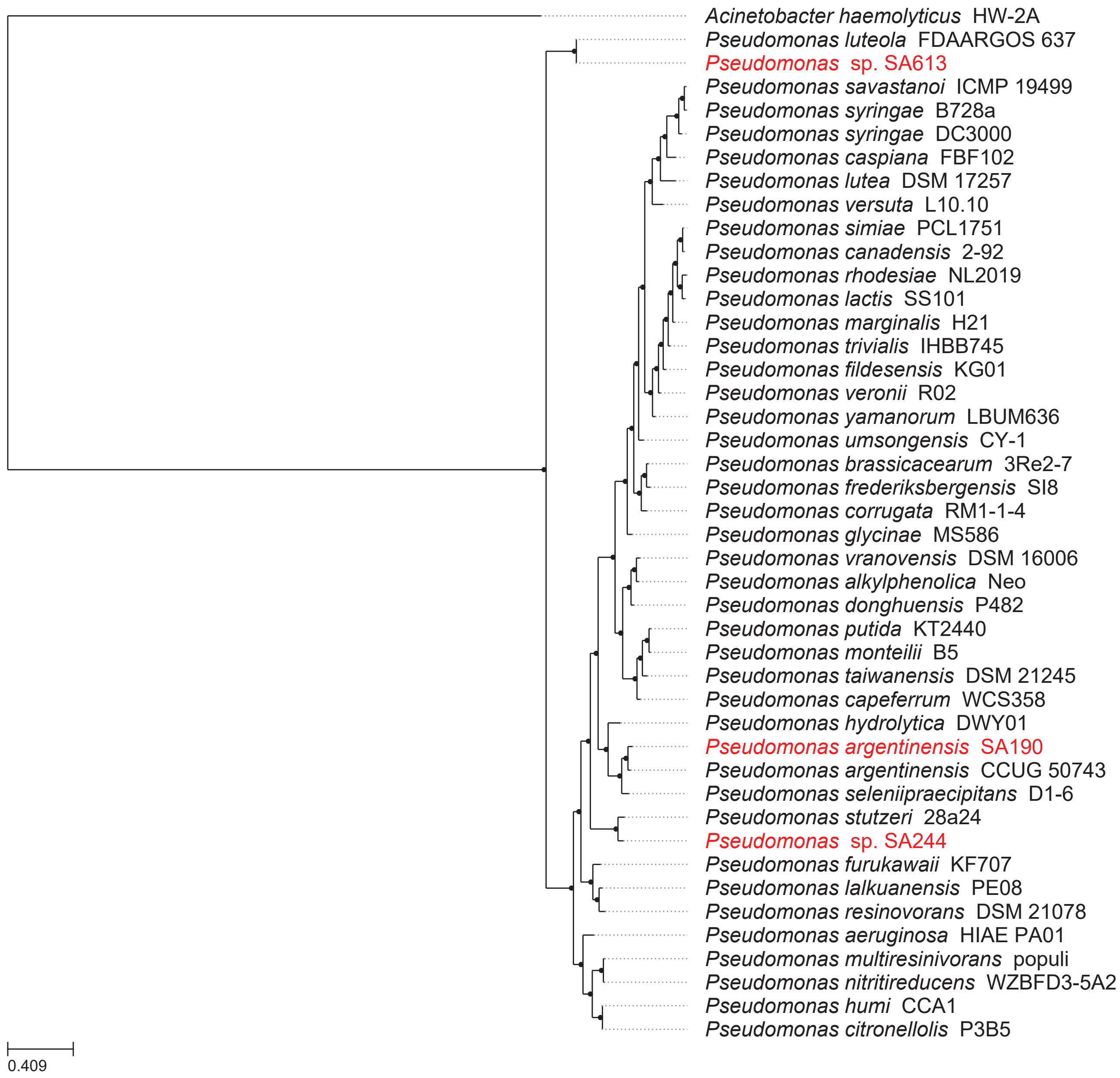

Acinetobacter haemolyticus HW-2A
Pseudomonas luteola FDAARGOS 637
Pseudomonas sp. SA613
Pseudomonas savastanoi ICMP 19499
Pseudomonas syringae B728a
Pseudomonas syringae DC3000
Pseudomonas caspiana FBF102
Pseudomonas lutea DSM 17257
Pseudomonas versuta L10.10
Pseudomonas simiae PCL1751
Pseudomonas canadensis 2-92
Pseudomonas rhodesiae NL2019
Pseudomonas lactis SS101
Pseudomonas marginalis H21
Pseudomonas trivialis IHBB745
Pseudomonas fildesensis KG01
Pseudomonas veronii R02
Pseudomonas yamanorum LBUM636
Pseudomonas umsongensis CY-1
Pseudomonas brassicacearum 3Re2-7
Pseudomonas frederiksbergensis SI8
Pseudomonas corrugata RM1-1-4
Pseudomonas glycinae MS586
Pseudomonas vranovensis DSM 16006
Pseudomonas alkylphenolica Neo
Pseudomonas donghuensis P482
Pseudomonas putida KT2440
Pseudomonas monteilii B5
Pseudomonas taiwanensis DSM 21245
Pseudomonas capeferrum WCS358
Pseudomonas hydrolytica DWY01
Pseudomonas argentinensis SA190
Pseudomonas argentinensis CCUG 50743
Pseudomonas seleniipraecipitans D1-6
Pseudomonas stutzeri 28a24
Pseudomonas sp. SA244
Pseudomonas furukawaii KF707
Pseudomonas lalkuanensis PE08
Pseudomonas resinovorans DSM 21078
Pseudomonas aeruginosa HIAE PA01
Pseudomonas multiresinivorans populi
Pseudomonas nitritireducens WZBFD3-5A2
Pseudomonas humi CCA1
Pseudomonas citronellolis P3B5
0.409

i

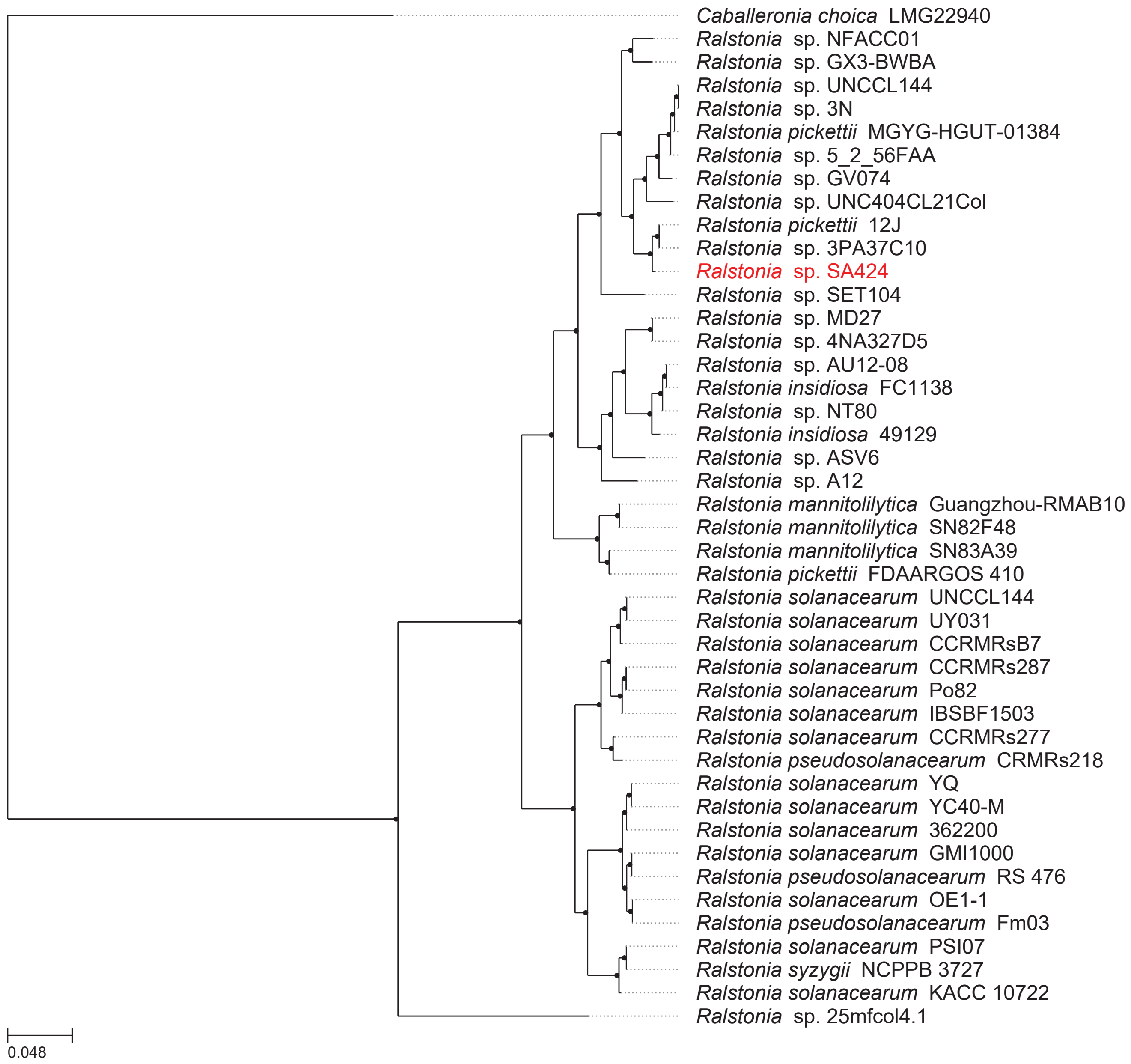
Caballeronia choica LMG22940
Ralstonia sp. NFACC01
Ralstonia sp. GX3-BWBA
Ralstonia sp. UNCCL144
Ralstonia sp. 3N
Ralstonia pickettii MGYG-HGUT-01384
Ralstonia sp. 5_2_56FAA
Ralstonia sp. GV074
Ralstonia sp. UNC404CL21Col
Ralstonia pickettii 12J
Ralstonia sp. 3PA37C10
Ralstonia sp. SA424
Ralstonia sp. SET104
Ralstonia sp. MD27
Ralstonia sp. 4NA327D5
Ralstonia sp. AU12-08
Ralstonia insidiosa FC1138
Ralstonia sp. NT80
Ralstonia insidiosa 49129
Ralstonia sp. ASV6
Ralstonia sp. A12
Ralstonia mannitolilytica Guangzhou-RMAB10
Ralstonia mannitolilytica SN82F48
Ralstonia mannitolilytica SN83A39
Ralstonia pickettii FDAARGOS 410
Ralstonia solanacearum UNCCL144
Ralstonia solanacearum UY031
Ralstonia solanacearum CCRMRsB7
Ralstonia solanacearum CCRMRs287
Ralstonia solanacearum Po82
Ralstonia solanacearum IBSBF1503
Ralstonia solanacearum CCRMRs277
Ralstonia pseudosolanacearum CRMRs218
Ralstonia solanacearum YQ
Ralstonia solanacearum YC40-M
Ralstonia solanacearum 362200
Ralstonia solanacearum GMI1000
Ralstonia pseudosolanacearum RS 476
Ralstonia solanacearum OE1-1
Ralstonia pseudosolanacearum Fm03
Ralstonia solanacearum PSI07
Ralstonia syzygii NCPPB 3727
Ralstonia solanacearum KACC 10722
Ralstonia sp. 25mfcol4.1
0.048

Fig. S5: Tomato salt stress assay. The salt stress assay was adapted from a protocol by Awlia et al., 2016. Pots with plants are soaked in a saline solution for about 30 minutes. As the WHC is always maintained at 60%, there is a certain volume of water already present in the substrate. When the saline solution enters through the bottom of the cups during the soaking, the salt concentration lowers as the solution is diluted by the water present in the substrate. The volume of saline solution that enters the pot is equal to the remaining 40% WHC plus extra air. This extra air volume is a result of the supersaturation by the soaking method which exceeds the amount predicted by 100% WHC. As the WHC returns to 60% due to evaporation and transpiration, the diluted saline solution will concentrate to a target concentration in the substrate.

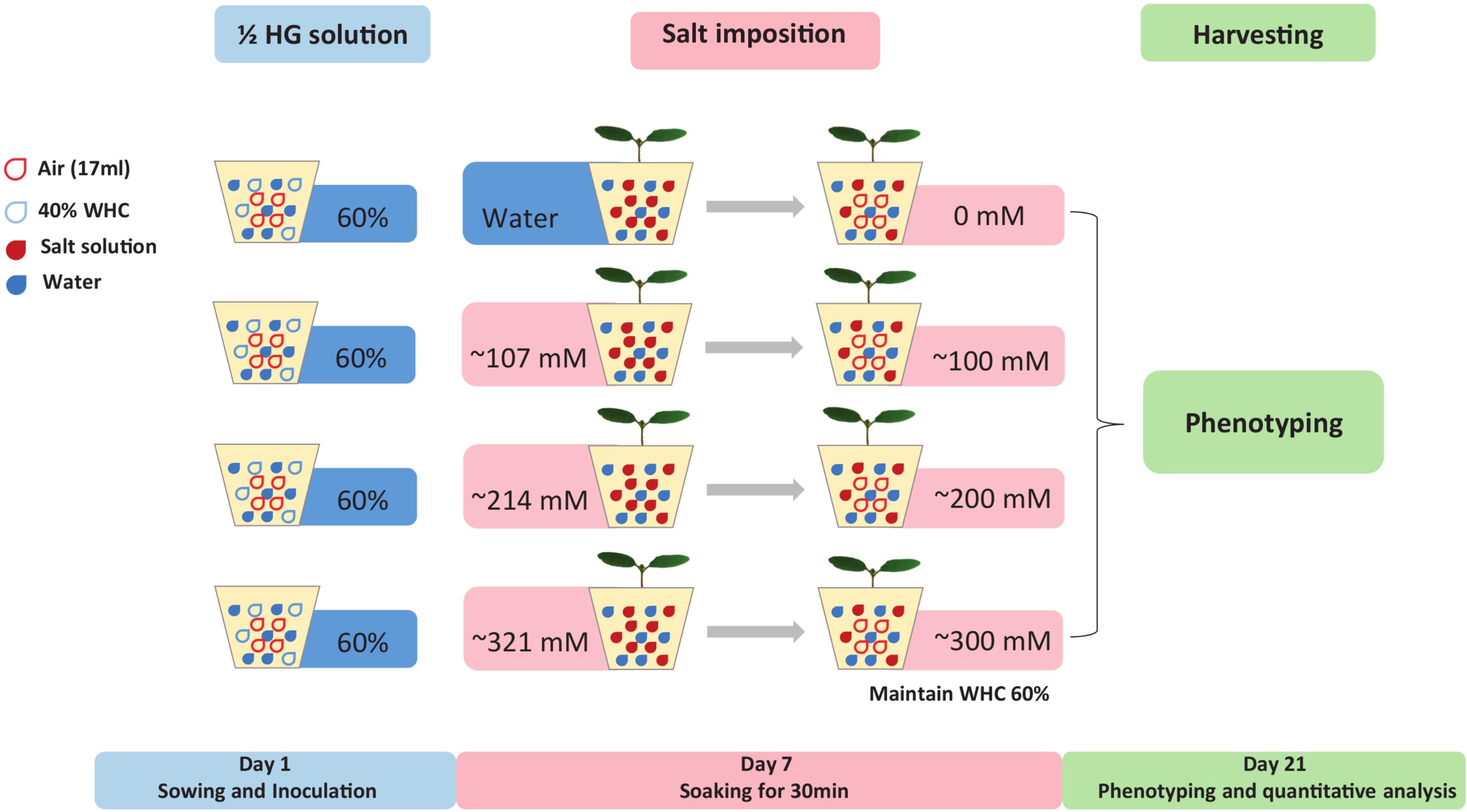

Fig. S6: Salt tolerance test of tomato plants grown with or without SynCom inoculation. Shoot dry weight of 21-day-old tomato plantlets exposed to 0, 100, 200, 300 mM NaCl (for 14 days) and inoculated with or without SynCom C. Plants were grown in the non-sterile substrate. An asterisk represents a statistically significant ($p < 0.05$) difference between the two inoculations per Student’s t-test.

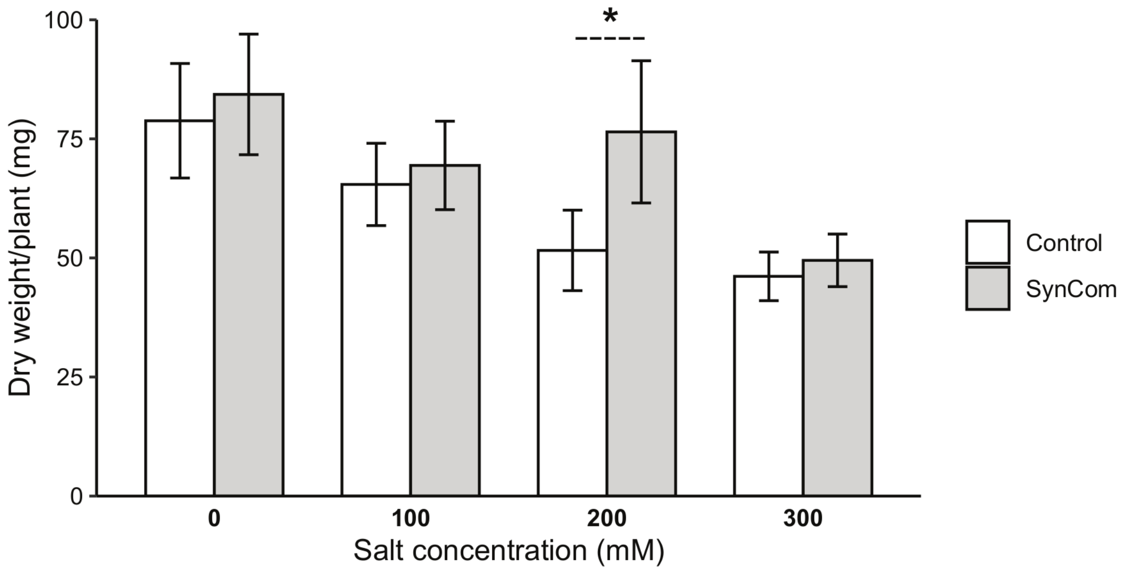

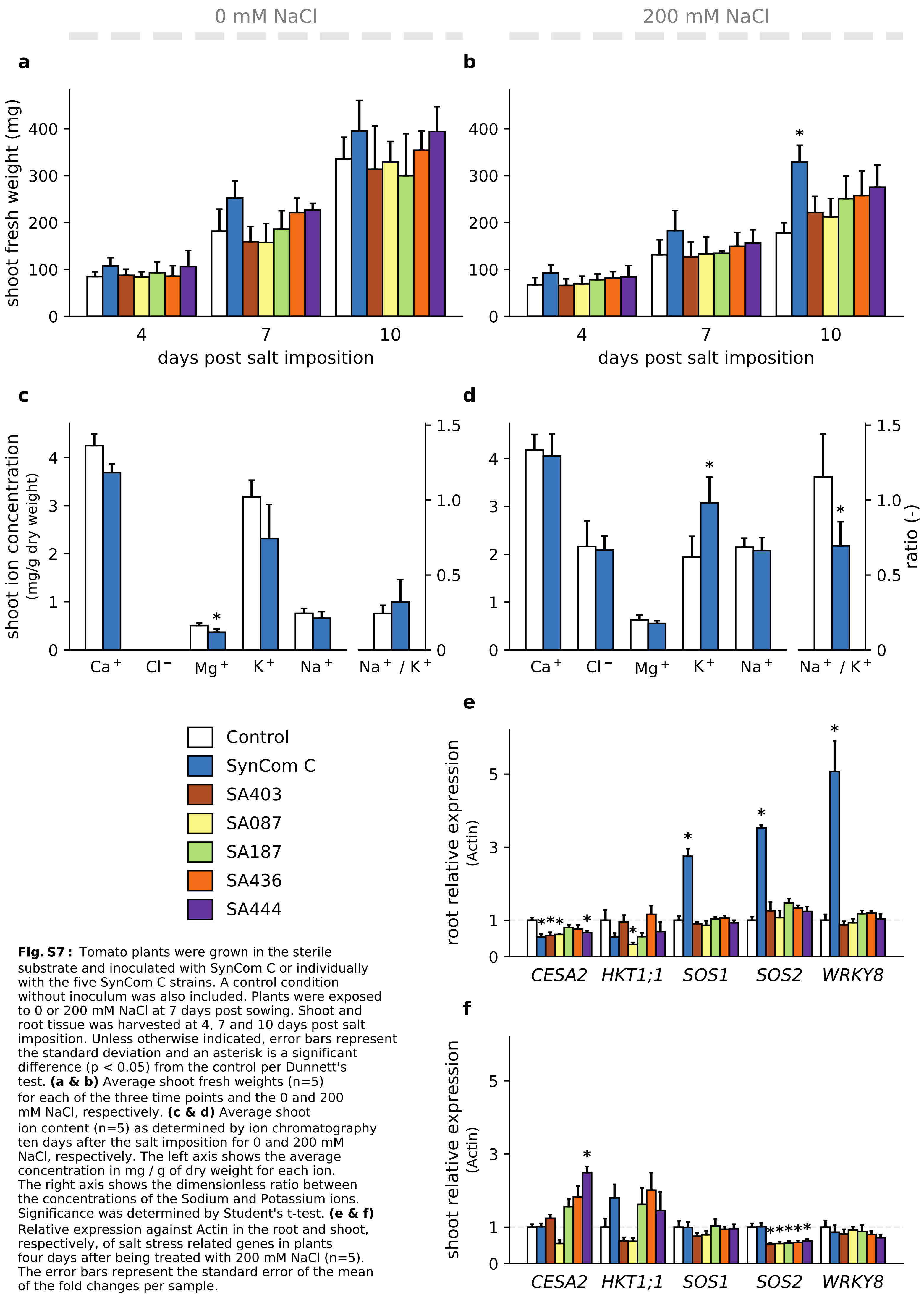

Fig. S7 : Tomato plants were grown in the sterile substrate and inoculated with SynCom C or individually with the five SynCom C strains. A control condition without inoculum was also included. Plants were exposed to 0 or 200 mM NaCl at 7 days post sowing. Shoot and root tissue was harvested at 4, 7 and 10 days post salt imposition. Unless otherwise indicated, error bars represent the standard deviation and an asterisk is a significant difference ($p < 0.05$) from the control per Dunnett's test. **(a & b)** Average shoot fresh weights (n=5) for each of the three time points and the 0 and 200 mM NaCl, respectively. **(c & d)** Average shoot ion content (n=5) as determined by ion chromatography ten days after the salt imposition for 0 and 200 mM NaCl, respectively. The left axis shows the average concentration in mg / g of dry weight for each ion. The right axis shows the dimensionless ratio between the concentrations of the Sodium and Potassium ions. Significance was determined by Student's t-test. **(e & f)** Relative expression against Actin in the root and shoot, respectively, of salt stress related genes in plants four days after being treated with 200 mM NaCl (n=5). The error bars represent the standard error of the mean of the fold changes per sample.

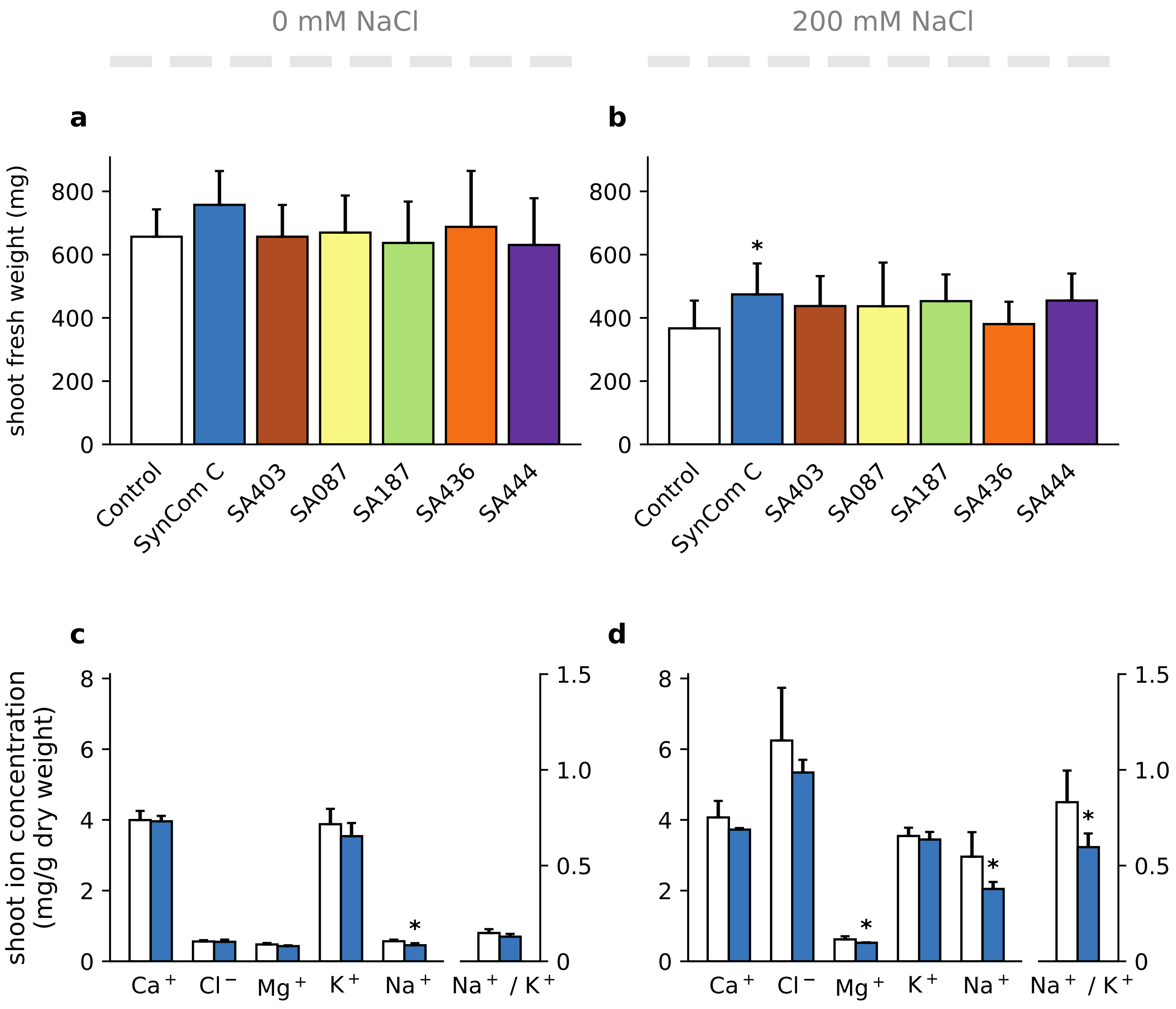

Fig. S8 : Tomato plants were grown in the non-sterile substrate and inoculated with SynCom C or individually with the five SynCom C strains. A control condition without inoculum was also included. Plants were exposed to 0 or 200 mM NaCl one week after sowing. Shoot tissue was harvested at ten days post salt imposition for fresh weight and ion content analysis. The standard deviation is shown by the error bars. **(a & b)** Average shoot fresh weights (n=15) for the 0 & 200 mM NaCl treated plants, respectively. An asterisk indicates a significant difference ($p < 0.05$) from the control per Dunnett's test. **(c & d)** Average shoot ion content as determined by ion chromatography for the 0 & 200 mM NaCl salt levels, respectively. Five random plants were selected from the control and SynCom C inoculated plants. The left axis shows the concentration in mg / g of dry weight for each ion. The right axis shows the dimensionless ratio between the concentrations of the Sodium and Potassium ions. An asterisk is a significant difference ($p < 0.05$) per Student's t-test.

Fig. S9: ASVs of Jizan strains which did not pass the detection criteria for the root colonization of tomato.

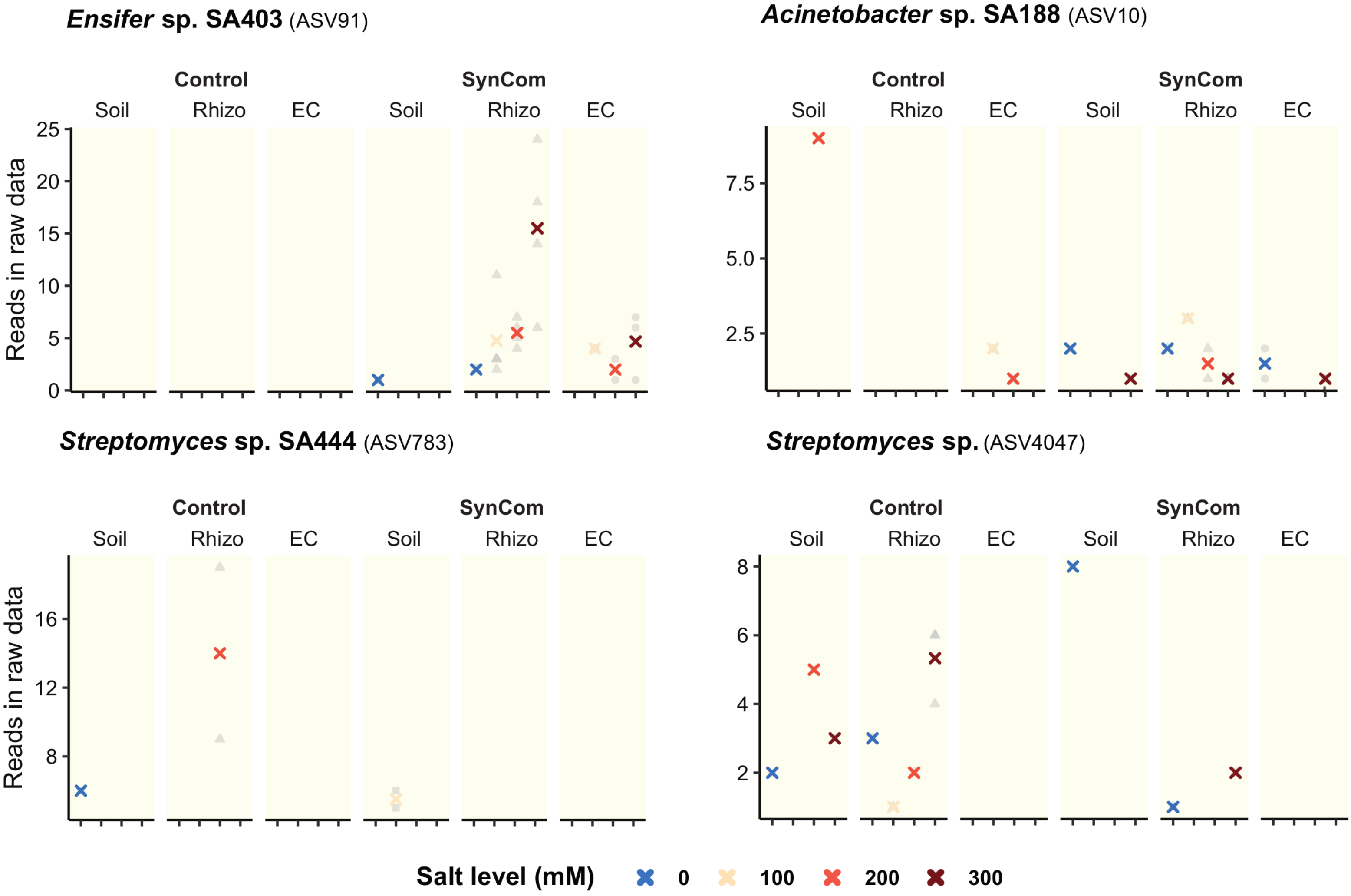

Fig. S10: Positive correlation between bacterial ASVs and salt concentration. Log2-transformed normalized read counts of ASVs found in the Rhizo (a) and EC (b) of tomato growing in the non-sterile substrate and inoculated with the Jizan SynCom. The ASVs that were enriched in any of the salt levels and with a positive correlation to the salt gradient are shown here. Crosses indicate mean log2-transformed normalized read counts of each ASV under each salt level. Blue crosses indicate ASVs matching a SynCom strain detected in this analysis.

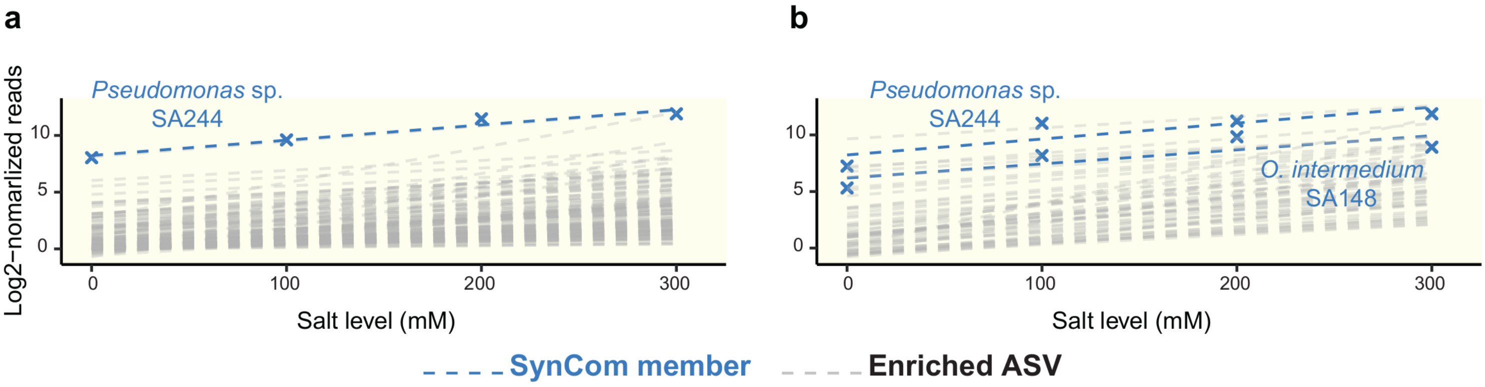

Supplement: Supplementary file 1 — Supplementary files [file 41396_2022_1238_MOESM1_ESM.pdf]
